# Synthesis of [^18^F]rufinamide as a radiotracer for epileptic brain imaging

**DOI:** 10.1039/d5ra09547f

**Published:** 2026-03-19

**Authors:** Vaibhav Pandey, Mohd. Faheem, Sanjay Gambhir, Manish Dixit

**Affiliations:** a Department of Nuclear Medicine, Sanjay Gandhi Postgraduate Institute of Medical Sciences Lucknow UP 226014 India dixitm@sgpgi.ac.in

## Abstract

Epilepsy is a multifactorial neurological disorder characterized by recurrent, unprovoked seizures. Although various diagnostic approaches are available, no single technique serves as a gold standard. Radiotracer-based positron emission tomography (PET) offers a non-invasive strategy for localizing epileptic foci. However, the currently available tracers have limited specificity and pharmacokinetic performance. In this work, report the radiosynthesis and preliminary evaluation of [^18^F]rufinamide as a potential PET tracer for epilepsy imaging. Microwave-assisted (µE) radiosynthesis (120 °C, 20 min) afforded quantitative conversion with a total synthesis time of 40 min from the end of bombardment (EOB), and a two-step procedure completed with 20 ± 5%, radiochemical yield and >95% radiochemical purity. The physicochemical profiling revealed a lipophilic nature (log *D*_7.4_) and high plasma protein binding (82.5 ± 2.1%). PET imaging and biodistribution studies in normal Wistar rats confirmed brain uptake and renal clearance. These findings demonstrate that [^18^F]rufinamide possesses favorable radiochemical and pharmacological characteristics, supporting its potential as a novel PET probe for non-invasive imaging of epileptic brain regions.

## Abstract 2

Epilepsy is a multifactorial neurological disorder characterized by recurrent, unprovoked seizures.

## Graphical abstract

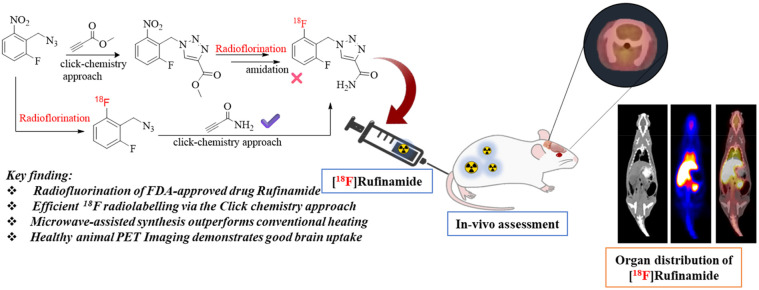

## 1. Introduction

Epilepsy is a complex neurological disorder that can occur due to unprovoked episodes of seizures. Globally, around 70 million people are affected, making it one of the most common neurological conditions.^1–3^ According to the WHO, more than 80% of epilepsy patients live in low and middle-income countries. Approximately 30% of cases remain poorly diagnosed or treatment-resistant, progressing to refractory epilepsy, for which surgical intervention is often considered the most suitable option.^4^ If the patients with epilepsy are adequately diagnosed and treated, around 70% of the cases can be controlled.^5^ Many FDA-approved anti-epileptic drugs (AEDs) control and manage epilepsy, but none are entirely medically fit for a broad spectrum. The diagnosis of epilepsy is also challenging, and this also accounts for poor and ineffective treatment with existing drugs. Rufinamide, approved by the European Commission and the US FDA in 2007 and 2008, respectively, exhibits broad-spectrum antiepileptic effects. Chemically, it is a triazole-based anticonvulsant that stabilizes neuronal excitability.^6–8^ The literature reports that rufinamide prolongs the inactivated state of voltage-gated sodium channels, thereby reducing excessive sodium-dependent action potential firing.^6–8^

Several studies have explored radiolabeled ligands targeting specific receptors for the imaging and diagnosis of epilepsy using PET/CT, particularly with carbon-11 (^11^C) and fluorine-18 (^18^F) radionuclides.^9,10^ The most common radiotracer for epileptic brain imaging is [^18^F]FDG ([^18^F]fluorodeoxyglucose), which measures regional cerebral glucose metabolism and lacks specificity. Some evidence suggests that hypometabolism may extend beyond the lesion area.^11^ Therefore, the use of [^18^F]FDG PET in the diagnosis of epilepsy remains questionable. The research focused on target-specific imaging agents, including GABA_A_, dopamine D2, NMDA, and SV2A-based radiotracers, as listed in Table 1.

**Table 1:** Representative PET radiotracers reported for brain imaging applications

Target	Radiotracer	Target	Radiotracer
D2/D3 receptor	[^18^F]-fallypride^12^	SV2A	[^11^C]UCB-J^21^
			[^18^F]-SynVesT-1 (ref. 21)
5-HT1A receptor	[^18^F]-FCWAY^13^	P-glycoprotein	[^11^C]-verapamil^22^
			[^11^C]-phenytoin^23^
mGluR5	[^11^C]-ABP688 (ref. 14)	Dopamine synthesis	[^18^F]FDOPA^24^
GABA_A_ receptor	[^11^C]/[^18^F]-FMZ^15^	Metabolic trapping	[^99m^Tc]ECD^25^
	[^123^I]-IMZ^16^		
	[^11^C]-Ro15-4513 (ref. 17)		
	[^11^C]Flumazenil^18^		
Serotonin synthesis	[^11^C]-AMT^19^	Dopamine D2 receptor	[^11^C]Raclopride^26^
AMPA receptors	[^11^C]-K-2 (ref. 20)	NMDA receptor	[^18^F]GE179 (ref. 27)

These radiotracers can identify more defined regions of abnormality within the epileptogenic foci and show greater sensitivity in extratemporal areas. Despite these advances, radiopharmaceutical synthesis often involves complex multistep protocols, requires advanced infrastructure, and demands rigorous quality control, all of which increase cost and hinder clinical accessibility.

The incorporation of fluorine-18 into rufinamide to synthesize [^18^F]rufinamide, while preserving its pharmacological properties. Several beneficial properties of rufinamide include potent antiepileptogenic effects and the absence of severe adverse effects. The complete molecular function of drug interaction remains elusive, and its precise mechanism in preventing seizures remains unclear. According to Drug Bank (https://www.drugbank.com), the half-life of rufinamide is 6–10 hours. These properties of rufinamide can be used to explore its potential for PET/CT imaging of epileptic regions. Gouverneur *et al.*^28^ (2017) reported Cu-mediated [^18^F]fluorination of various boronic ester-containing heterocyclic compounds, including the boronic ester precursor of [^18^F]rufinamide. This study focuses on the core synthetic strategy with radiochemical conversion (RCC), which yielded 17–19% (*n* = 3) for [^18^F]rufinamide. The present study utilizes a modular azide–alkyne cycloaddition strategy. This approach enables the comprehensive radiopharmaceutical evaluation of [^18^F]rufinamide, including reaction optimization, physicochemical profiling, and *in vivo* assessment through PET imaging performed in normal Wistar rats. These studies were performed to confirm the blood–brain barrier permeability of [^18^F]rufinamide through its biodistribution and brain uptake.

## 2. Materials and methods

### 2.1 General methods

All solvents and reagents were purchased from Sigma-Aldrich and stored under dry conditions. All reactions were carried out using oven-dried glassware. The radiolabelling *via* nucleophilic aromatic substitution reaction (SNAr) was performed both in conventional heating and in a microwave heating (CEM Corporation) using 10 mL pressure tube.^29–31^ The fluorine-18 was obtained by irradiation of protons on [^18^O]OH_2_ (ABX Advance Med. Compound, GmbH) *via* the ^18^O(p,n)^18^F nuclear reaction using Medical Cyclotron (Sumitomo Heavy Industries Ltd, Japan). The irradiation was performed at a current of 30 µA for 10 minutes. At the end of bombardment, activity typically ranged from 5.55 to 9.25 GBq was received. The radiolabeling was performed in dry DMSO as the solvent under various conditions, including different times, temperatures and heating methods. The radiochemical conversion (RCC) and radiochemical purity (RCY) were calculated using radio thin-layer chromatography (abbreviated as rTLC; Miniscan Pro, Eckart & Ziegler Europe GmbH, Germany) and radio high-performance liquid chromatography (abbreviated as rHPLC; Agilent Technologies, Germany). All radiochemical yields were decay corrected to the [^18^F]fluoride activity measured at the start of synthesis. The reported reaction conditions gave the highest yields obtained by manual radiosynthesis.

### 2.2 Experimental section

#### 2.2.1 General synthesis

**Scheme 1:**
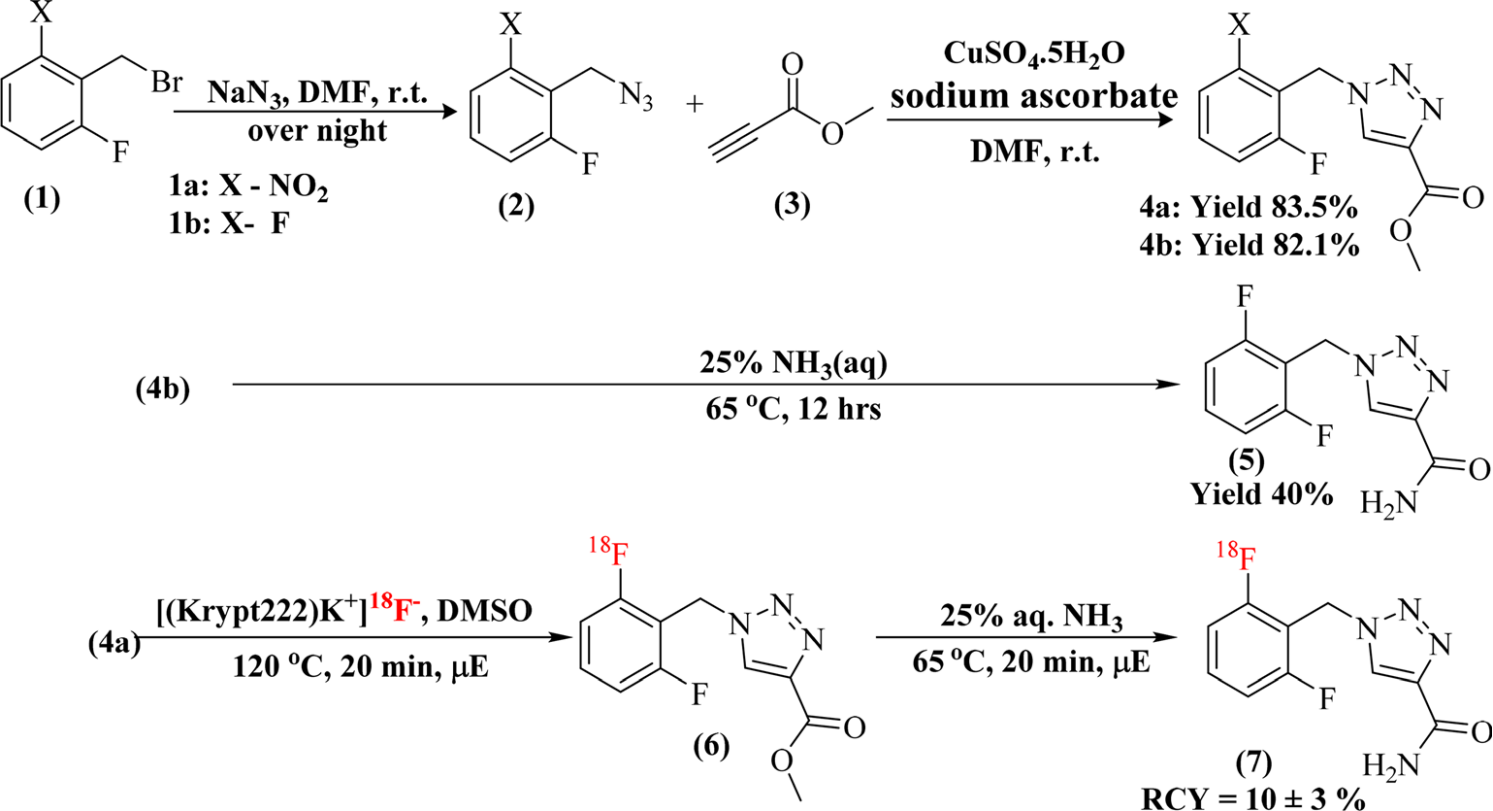
First radiosynthetic route for the preparation of [^18^F]rufinamide.

**Scheme 2:**
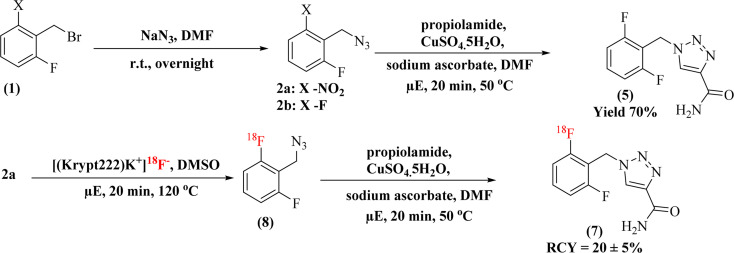
Second radiosynthetic route for the preparation of [^18^F]rufinamide.

The synthesis procedure was adopted from the literature with slight modifications.^32^ In dry DMF (10 mL), a solution of 2-nitro/fluoro-6-fluorobenzyl bromide 1a–b (500 mg, 2.13 mmole, 1 eq.) and sodium azide (153 mg, 2.35 mmole, 1.1 eq.) was stirred at room temperature overnight. After the reaction was completed, the solution was diluted with cold water and extracted with ethyl acetate. The ethyl acetate layer was washed with brine, dried over anhydrous sodium sulphate, and concentrated under reduced pressure to afford the corresponding azide 2a–b as a yellow oily intermediate. Subsequently, compound 2a–b (550 mg, 2.85 mmole, 1.2 eq.) was resuspended in dry DMF (5 mL) and methyl propiolate 3 (470 mg, 2.38 mmole, 1 eq.) was added to the reaction before the addition of copper sulfate pentahydrate (118 mg, 0.47 mmole, 0.2 eq.) and sodium ascorbate (188 mg, 0.95 mmole, 0.4 eq.) for the formation of the desired triazole ring. The reaction mixture was stirred at room temperature, and TLC was used to monitor the reaction progress. After the reaction was complete, the mixture was poured into ice water, and the precipitate was collected. It was washed with a small amount of cold ethyl acetate to afford the crude product, which was purified again by column chromatography (ethyl acetate : hexane, 3 : 2) to obtain the purified product (Schemes 1 and 2).

#### 2.2.2 Synthesis of methyl-1-(2-nitro-6-fluorobenzyl)-1*H*-1,2,3-triazole-4-carboxylate (4a)

Yield compound 4a, off white colour with 83% overall yield, ^1^H NMR (400 MHz, DMSO-d_6_) *δ* 8.85 (s, 1H), 8.04–7.97 (m, 1H), 7.78 (dd, *J* = 8.4, 4.9 Hz, 2H), 5.88 (s, 2H), 3.82 (s, 3H). ESI + APCI (M + H^+^) *m*/*z*: calc. 281.2, found 280.9.

#### 2.2.3 Synthesis of methyl-1-(2,6-difluorobenzyl)-1*H*-1,2,3-triazole-4-carboxylate (4b)

Yield compound 4b (82%) as a white solid (4b), ^1^H NMR (400 MHz, DMSO-d_6_) *δ* 8.84 (s, 1H), 7.52 (tt, *J* = 8.6, 6.7 Hz, 1H), 7.18 (t, *J* = 8.1 Hz, 2H), 5.74 (s, 2H), 3.83 (s, 3H). ESI + APCI (M + H^+^) *m*/*z*: calc. 254.2, found 254.0.

#### 2.2.4 Synthesis of rufinamide (standard) (5)

Route 1: the compound 4b (100 mg, 0.395 mmol) was treated with 25% aqueous methanolic ammonia (5 mL) at 65 °C for 12 hours to convert the ester group to an amide, yielding crude rufinamide (5) (Scheme 1). The crude was purified by silica gel column chromatography using dichloromethane (DCM) with 1 to 5% of methanol (MeOH) as a mobile phase to obtain the purified compound 5 with 40% yield. ^1^H NMR (400 MHz, DMSO-d_6_) *δ* 8.41 (s, 1H), 7.75 (s, 1H), 7.66–7.32 (m, 2H), 7.25 (t, *J* = 8.1 Hz, 2H), 5.65 (s, 2H). LC-MS (M + H^+^) *m*/*z*: calc. 239.2, found 239.1.

Route 2: in a round-bottom flask, compound 2b (120 mg, 0.7 mmole, 1.2 eq.) was dissolved in dry DMF (2 mL), and propiolamide (50 mg, 0.6 mmole, 1 eq.) was added. After that, sodium ascorbate (47 mg, 0.24 mmole, 0.4 eq.) and copper sulfate pentahydrate (0.30 mg, 0.12 mmole, 0.2 eq.) were sequentially added to the reaction mixture to perform the click reaction in microwave conditions at 50 °C (Scheme 2). After the reaction was complete, the crude mixture was poured into ice. The precipitate obtained was washed with a small amount of cold ethyl acetate to afford the crude product, which was then purified by silica gel column chromatography using DCM with 1–5% MeOH as the mobile phase to obtain compound 5 with 70% yield as a white solid.

^1^H NMR (400 MHz, DMSO-d_6_) *δ* 8.54 (s, 1H), 7.83 (s, 1H), 7.58–7.44 (m, 2H), 7.19 (t, *J* = 8.1 Hz, 2H), 5.72 (s, 2H). ESI + APCI (M + H^+^) *m*/*z*: calc. 239.2, found 239.1.

### 2.3 Radiochemistry

The radiolabelling *via* an SNAr reaction was performed using a standard 18F chemistry protocol, as documented in the literature.^29–35^ The received F-18 was purified using a SepPak carbonated QMA cartridge and eluted with a Kryptofix (0.058 mmole) and potassium carbonate (0.041 mmole) solution to form the [(Krypt222)K^+^]^18^F^−^ complex. The complex was dried with acetonitrile *via* azeotropic at 80 °C to form anhydrous [(Krypt222)K^+^]^18^F^−^. The compound 4a (5.0 mg) was dissolved in anhydrous DMSO (100 µL) in a V-shaped vial, and the activity (0.37–3.7 GBq) was added. The reaction vial was heated at various temperatures (80–150 °C) and times (3–20 min). The heating was performed using both conventional and microwave methods. The labelled compound RCC and RCY was assessed both *via* rTLC and HPLC.

### 2.4 Quality control

Rufinamide as standard and [^18^F]rufinamide were analyzed using rTLC and analytical HPLC equipped with Eclipsed plus C18 (250 × 4.6 mm, 5 µm) with a variable wavelength detector (VWD). The column was maintained at room temperature, and the mobile phase consisted of (A) acetonitrile and (B) water containing 0.1% trifluoroacetic acid (TFA), in a 90 : 10 (v/v) ratio with a flow rate of 0.7 mL min^−1^. The radiation signal was analyzed by a NaI(Tl)-based radioactive detector (EZScan, Inc., USA).

### 2.5 Protein binding study (%PPB)

The percentage of protein binding^36^ was assessed by incubating [^18^F]rufinamide (∼4.0 MBq) in 450 µL of freshly prepared human serum and albumin at 37 °C for up to 1 h, respectively. After 1 h, 450 mL of acetonitrile (1 : 1 v/v) was added to both human serum and albumin to precipitate all proteins. The samples were vortexed for 1 minute, then centrifuged for 6 minutes at 3000 rpm. The resulting pellet (protein-bound fraction) and supernatant (free fraction) were collected separately, and their radioactivity was measured using a γ-counter. The PPB was performed in triplicate (*n* = 3), and results are expressed as mean ± SD, bar graph was generated by GraphPad. The percentage of protein binding was calculated as follows
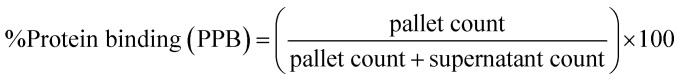
where: pellet counts = bound fraction; supernatant counts = free fraction.

### 2.6 Distribution coefficient (log *D*_7.4_) measurements

The distribution coefficient (log *D*_7.4_) was calculated using the shake-flask method.^36^ Briefly, [^18^F]rufinamide (∼4.0 MBq) activity was added to a vial containing 500 µL of *n*-octanol and 500 µL of phosphate buffer (0.1 M, pH 7.4). The mixture was shaken for 30 minutes, then centrifuged at 3000 rpm for 6 minutes. A sample of 0.1 mL *n*-octanol and 0.1 mL buffer was taken to a separate vial, and activity was counted *via* a gamma counter. The distribution coefficient measurements were performed in triplicate (*n* = 3), and results are expressed as mean ± SD, bar graph was generated by GraphPad. The log *D*_7.4_ was calculated using the following equation:
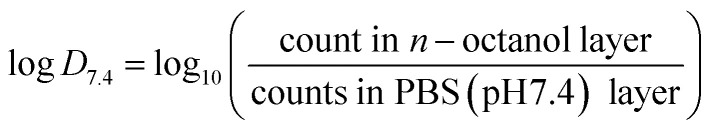


### 2.7 PET/CT imaging

All animal procedures were performed in accordance with the Guidelines for the Care and Use of Laboratory Animals of Sanjay Gandhi Postgraduate Institute of Medical Sciences (SGPGIMS), Lucknow, India, and were approved by the Institutional Animal Ethics Committee (Protocol No. 09-2018–2022). Healthy Wistar rats, aged 6–8 weeks, weighing 250–300 grams, were maintained in a sterile environment for the experiment. Local anaesthesia^37,38^ was induced in normal Wistar rats *via* the peritoneal and maintained for 120 min. The body temperature was maintained at 37 °C throughout the experiment. The rat was injected with ∼13.69 MBq of [^18^F]rufinamide, (3.7 MBq gram^−1^). The PET scans were recorded at different time intervals (0–120 min). The PET/CT scan was performed using a Biograph mCT S(64) – 3R PET/CT scanner (Siemens Medical Solutions, USA, Inc.), and images were recorded at different time intervals. The PET/CT scan was set for 1.5 minutes per bed. A total of two BEDs were performed to complete the whole-body scan. The image files were processed using the Syngo.*via* software (Siemens Medical Solution, USA). In processing, the defined organ circle of 3D regions and maximum Standardized Uptake Values (Lean Body Mass) [SUVmax(lbm)] were calculated. Excel generated the Organ SUVmax/time–activity curves. *Ex vivo* bio-distribution was calculated for kidneys, lungs, heart, liver, spleen, pancreas, small intestine, stomach, muscle, and blood, and tumour samples were dissected. Radioactivity was determined in a sodium iodide detector. The results were expressed as a percentage of the injected dose per gram. The SUVmax measurements were performed triplat (*n* = 3), and results are expressed as mean ± SD.

## 3. Result and discussion

### 3.1 Chemistry & radiochemistry

**Scheme 3:**
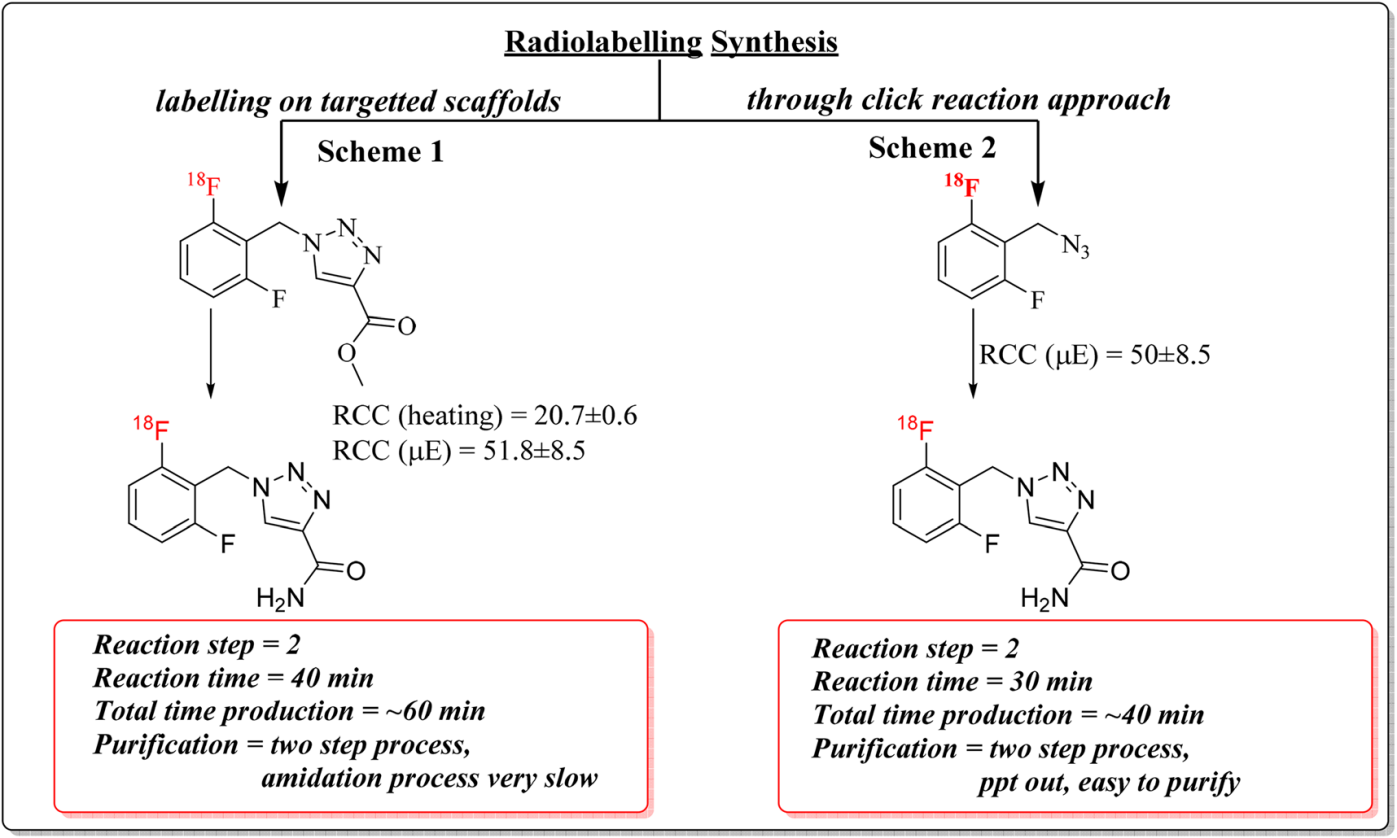
Overview of the overall radiolabelling procedures and corresponding radiochemical outcomes for [^18^F]rufinamide.

In this study, two radiolabelling strategies were developed and evaluated for the synthesis of [^18^F]rufinamide, as summarized in Scheme 3. Both methods involved SNAr-mediated ^18^F-fluoride incorporation but diverged in the subsequent chemistry to produce the final product.

#### 3.1.1 Route 1: SNAr radiolabelling followed by amidation

In the first approach, radiolabeling of precursor 4a was performed using an established ^18^F-SNAr protocol^29–35^ (Scheme 1). The active F-18 complex [(Krypt222)K^+^]^18^F^−^ was prepared with Kryptofix and potassium carbonate, then dried azeotropically in anhydrous acetonitrile at 80 °C. Precursor 4a (5.0 mg) dissolved in 100 µL of anhydrous DMSO was added to the dried complex (activity range: 0.37–3.7 GBq).

**Fig. 1:**
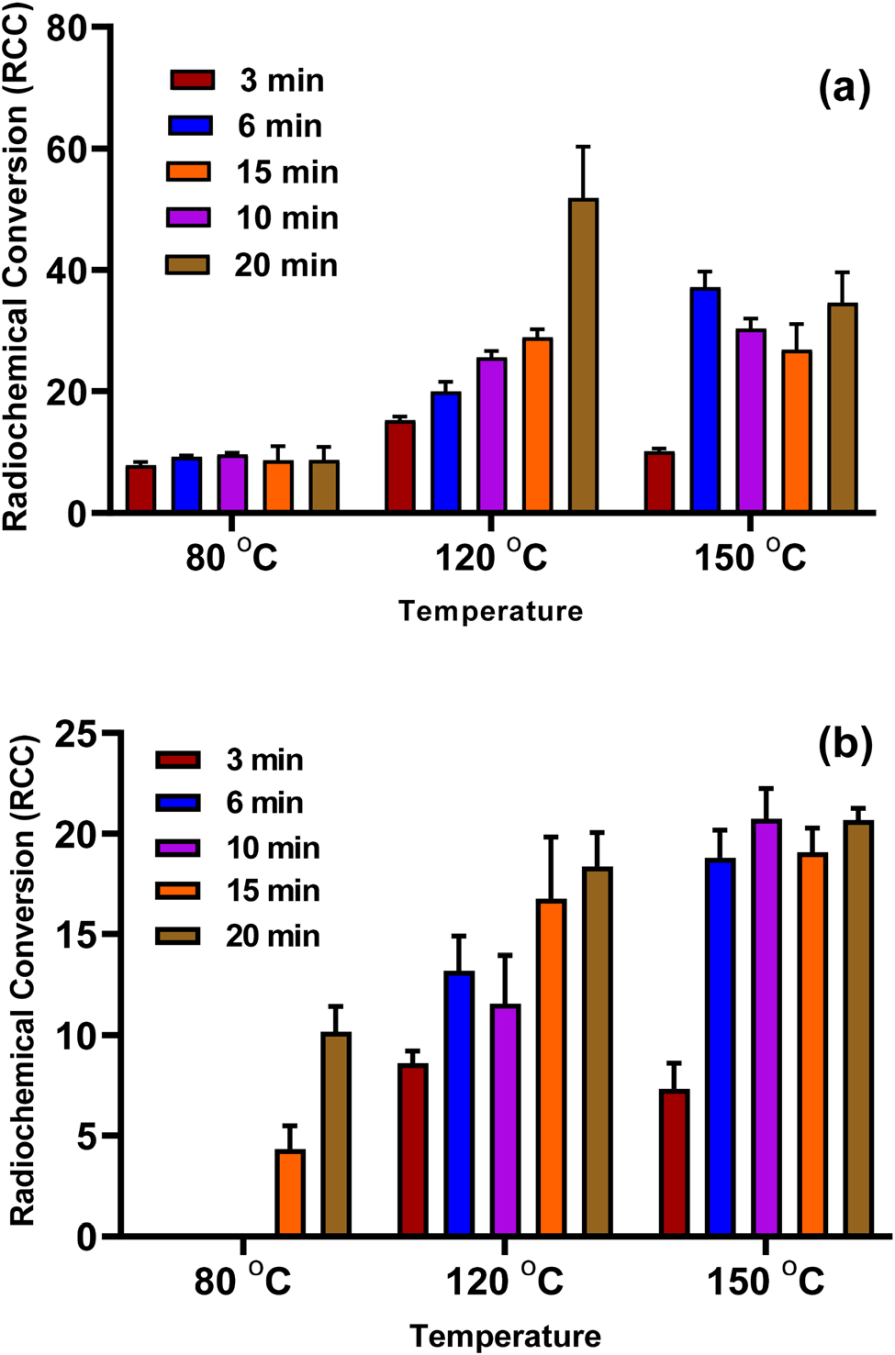
Radiochemical conversion (RCC) of [^18^F]rufinamide determined by radio-TLC analysis under different reaction conditions. Bar graphs represent RCC values calculated from radioactivity associated with product and precursor peaks. (a) RCC obtained at varying temperatures and reaction times under microwave heating. (b) RCC obtained at varying temperatures and reaction times under conventional heating. Data are presented as mean ± SD (*n* = 3).

**Fig. 2:**
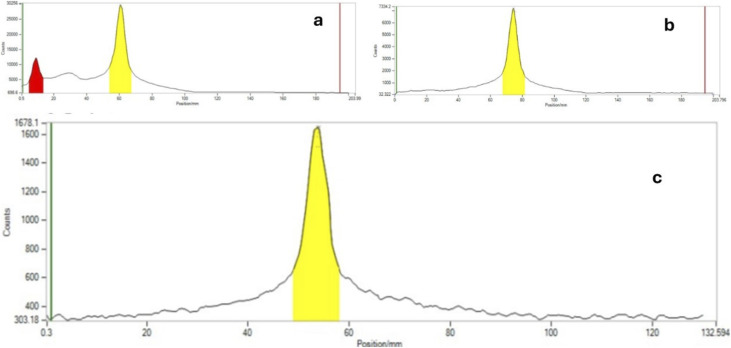
Radio thin layer scanner graph of first route product and intermediate (a) crude mixture of [^18^F]triazole ester, (b) purified radio scanner graph of [^18^F]triazole ester, and (c) [^18^F]rufinamide radiogram.

The reaction conditions were systematically optimized over a range of temperatures (80–150 °C) and times (3–20 minutes) using both conventional and microwave heating. Progress was monitored by silica TLC (methanol : water, 9 : 1) and quantified using a radio-TLC scanner. Microwave heating at 120 °C for 20 minutes yielded the highest radiochemical conversion (RCC) of 51.8 ± 8.5%, whereas conventional thermal conditions resulted in an RCC of 20.7 ± 0.6% (Fig. 1). The radiolabelled crude mixture was purified over a preconditioned SepPak C18 cartridge. The crude mixture dilute with 1 mL of water and load onto a C-18 cartridge, again washed with 20 mL of water to remove unreacted free ^18^F-fluoride. The ^18^F-intermediate (6) was eluted with methanol and confirmed by rTLC scanner (Fig. 2).

The intermediate (6) was subsequently treated with 25% aqueous ammonia solution (1.0 mL) at 65 °C for 20 minutes in a sealed pressure tube to effect amide formation, yielding crude [^18^F]rufinamide. A second SepPak C18 Plus cartridge was used for initial clean-up, with the product eluted in 0.5 mL ethanol, followed by final purification *via* semi-preparative reverse-phase HPLC (Phenomenex Luna, 5 µm, C18(2), 100 Å, 250 × 10 mm; mobile phase: water/1% TFA : acetonitrile, 50 : 50 v/v). The overall synthesis time was approximately 60 minutes, with a decay-corrected radiochemical yield (RCY) of ∼10 ± 3% (100–150 MBq). The rTLC analysis in methanol and water (9 : 1) confirmed the identity of the final product, with a retention factor (*R*_f_) of 0.275 for compound 5 (Fig. 2). Although [^18^F]rufinamide was successfully produced using this route, several practical issues were identified. The amidation step is naturally slow and results in low conversion efficiency in a radiosynthetic process, where the short half-life of ^18^F (*t*_1_/_2_ = 109.8 min) puts tight limits on total production time. The low radiochemical yield made purification difficult, making this method less suitable for routine production.

#### 3.1.2 Route 2: SNAr radiolabelling followed by click chemistry

To overcome issues of route 1, an alternative two-step sequence was developed that remove the amidation step entirely, instead coupling radiolabelled azide intermediate 8 with propiolamide *via* copper-catalysed azide–alkyne cycloaddition (CuAAC click chemistry) (Scheme 2).

The SNAr step was carried out with identical conditions to route 1, using precursor 2a (5.0 mg) in anhydrous DMSO (100 µL) at 120 °C for 20 minutes at 100 W under microwave irradiation, affording ^18^F-labelled azide intermediate 8. The crude mixture was loaded onto a SepPak C18 Plus cartridge, washed with 20 mL of water to remove free [^18^F]fluoride, and the trapped intermediate was eluted with dry DMF with minimum volume (∼500 µL). The radiolabelling was confirmed by rTLC and RCC was calculated. The RCC was found 50 ± 8.5% (Scheme 3).

After elution, the click reaction was carried out directly on the isolated [^18^F]azide by adding propiolamide (1 eq.), copper sulfate pentahydrate (0.2 eq.), and sodium ascorbate (0.4 eq.). The reaction was performed under microwave conditions at 50 °C for 10 minutes at 100 W. Complete consumption of the azide and formation of the 1,2,3-triazole scaffold, consistent with CuAAC regioselectivity reported in the literature, was indicated by the precipitation of the product upon cooling.

**Fig. 3:**
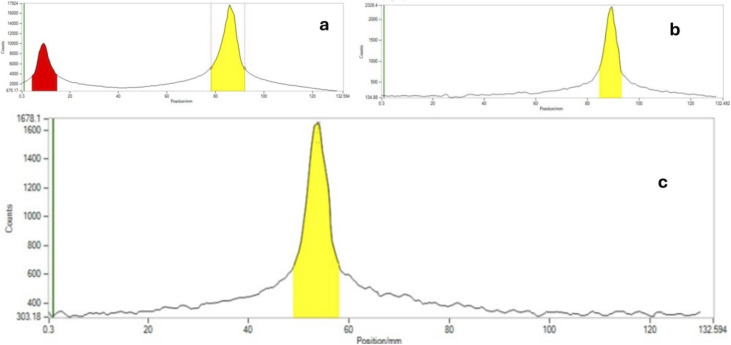
Radio-TLC chromatograms obtained during the second radiosynthetic route. (a) Crude reaction mixture containing [^18^F]azide. (b) Purified [^18^F]azide after isolation. (c) Radiochromatogram of the final product [^18^F]rufinamide.

This precipitate was collected on a 0.22 µm membrane filter, washed with 10 mL of water to remove other impurities, and [^18^F]rufinamide was recovered by elution with warm ethanol. In this methodological approach, a minor limitation was also observed, the challenging solubility of the precipitated product during hot chemistry within a limited time scale. The dissolution of the precipitate in hot chemistry conditions requires a larger volume of warm ethanol for the collection of [^18^F]rufinamide. The route-2 afforded a substantially improved decay-corrected radiochemical yield (RCY) of ∼20 ± 5% (250–400 MBq) with a radiochemical purity >95%, and the total synthesis time was reduced to approximately 40 minutes, a meaningful efficiency gain in ^18^F-radiochemistry. The rTLC analysis confirmed the radiolabelling distinct retention factors for the [^18^F]-azide intermediate (*R*_f_ = 0.426), free ^18^F-fluoride (*R*_f_ = 0.04), and the final [^18^F]rufinamide product (*R*_f_ = 0.265) (Fig. 3).

### 3.2 Quality control and analytical characterisation

**Fig. 4:**
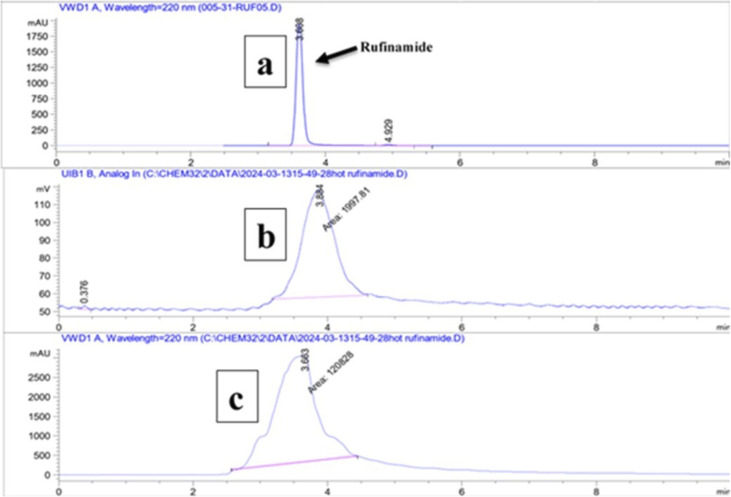
HPLC UV chromatogram along with radiation peak of [^18^F]rufinamide (a) chromatogram of standard rufinamide (b and c) chromatogram of radiation and UV spectra respectively.

The radiolabelling was characterized by radio-HPLC (rHPLC) and radio-TLC (rTLC). The mobile phase was used methanol and water (9 : 1) for rTLC, and acetonitrile with water-TFA 1% (90 : 10) for rHPLC, respectively. The radio scanner TLC data of [^18^F]azide, along with [^18^F] free fluoride, show retention factors (*R*_f_) 0.426 & 0.04, respectively, whereas the final compound [^18^F]rufinamide has an *R*_f_ = 0.265 (Fig. 3). The HPLC UV spectra of cold rufinamide indicate *t*_R_ = 3.6 min. The labelled [^18^F]rufinamide UV peak shows *t*_R_ = 3.8 min along with a radiation peak, which appears at 3.8 minutes, indicating the successful synthesis of [^18^F]rufinamide (Fig. 4).

In Scheme 2, the reaction is a straightforward method for synthesis, resulting in less time-consuming total production time, and provides a means for quality assurance analysis. In the final step, propagate the literature support that the complete consumption of azide results in a triazole skeleton through a click reaction. The drawback is that the precipitation is a good sign for purification, but in hot chemistry, further dissolution of this precipitate is less. More warm ethanol is used to dissolve the precipitate. After successfully radiolabelling and characterization, an *in vitro* experiment for the evaluation of the physicochemical properties of [^18^F]rufinamide was performed. The distribution coefficient (log *D*_7.4_) indicates the lipophilicity of a compound. Most of the count comes from the octanol portion as compared to PBS. The log *D*_7.4_ value is 0.72 ± 0.03 (lipophilic range; see SI document).

The percentage of plasma protein binding (%PPB) was evaluated in albumin and freshly prepared human serum. The percentage of PPB in human serum is 82.5 ± 2.1% (*n* = 3), whereas in albumin, it is 76.6 ± 1.1% (*n* = 3). After an *in vitro* study and quality assurance of the radiolabelled drug, an *in vivo* experiment was performed by injecting ∼13.69 MBq (3.7 MBq gram^−1^) in isotonic NaCl solution (0.9%) through the intravenous route through tail, and PET/CT imaging procedures were performed to acquire the imaging data.

### 3.3 PET imaging

**Fig. 5:**
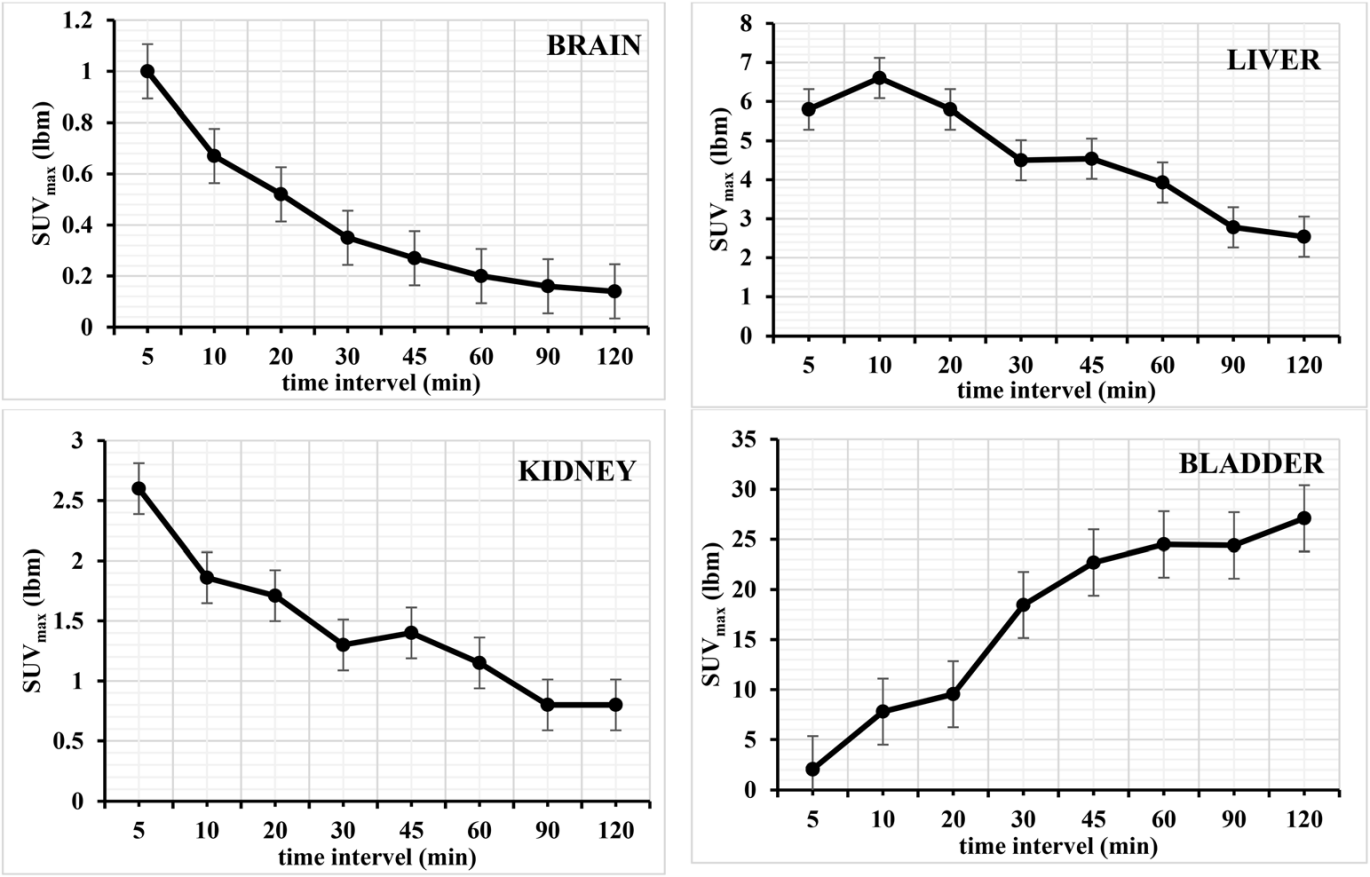
Time–activity curves showing the kinetic distribution of [^18^F]rufinamide in healthy Wistar rats. SUVmax (lbm) are plotted *vs.* time for brain, liver, kidney, and urinary bladder. Data are presented as mean ± SD (*n* = 3).

**Fig. 6:**
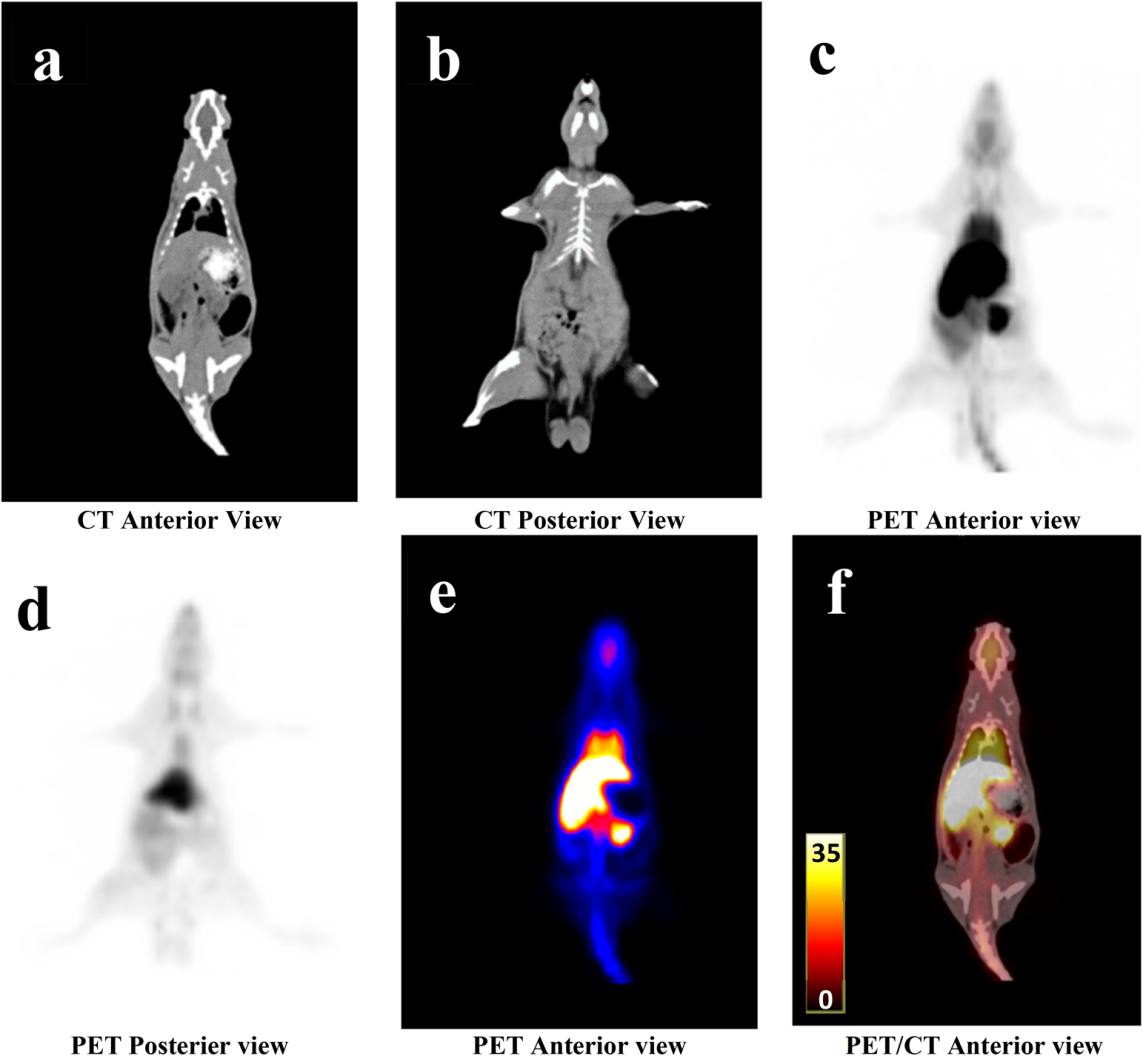
Representative PET/CT images of healthy Wistar rats acquired at 5 min post-injection (p.i.) of [^18^F]rufinamide. (a) CT image, anterior view. (b) CT image, posterior view. (c) Maximum intensity projection (MIP) PET image, anterior view. (d) Maximum intensity projection (MIP) PET image, posterior view. (e) Whole-body PET image showing maximum standardized uptake values (SUV), anterior view. (f) PET/CT fused image illustrating whole-body distribution of [^18^F]rufinamide and corresponding SUVmax, anterior view. Scale bar = 35 SUVmax/lbm.

The animal was positioned in the field of view of the PET/CT scanner immediately after the administration of [^18^F]rufinamide injection. Whole-body PET/CT images were acquired using a 2-bed position protocol, with a scan duration of 1.5 minutes per bed position. The PET/CT images were acquired between (05–120 minutes) after injection of [^18^F]rufinamide. The images were reconstructed and analysis was performed using Syngo.via software (Siemens Medical Solutions, USA, Inc.). The maximum intensity projection (MIP) and SUVmax (lbm) graphs are shown in Fig. 5 and 6.

The PET/CT images showed that the majority of the administered [^18^F]rufinamide activity was retained in the liver, with limited uptake in the brain. Based on SUV analysis, the brain shows an uptake of 1.5 ± 0.5 SUV, whereas the liver showed 5.8 SUV immediately after injection (5 min). Brain uptake gradually decreased over time, reaching 0.14 SUV at 120 min (see SI). The PET/CT data further indicated predominant hepatic retention at 5 min post-injection. Hepatic SUV declined over the following 120 min to 2.54 ± 0.5 (Fig. 5), followed by increased intestinal activity (see SI). These findings suggest preferential hepatobiliary elimination of [^18^F]rufinamide. In addition, renal excretion was also observed, as the radiotracer passed through the kidneys and accumulated in the urinary bladder. Accordingly, bladder SUV increased progressively from 5 min to 120 min (Fig. 5).

The images show that very low uptake (negligible) in other organs such as the lung, spleen, pancreas, bone marrow and muscle. The detailed view of the tracer kinetics is reported in the SI document. The present study was conducted in normal Wistar rats, the measured SUVmax (lbm) values reflect non-specific physiological brain uptake and tracer pharmacokinetics rather than target-specific binding.

The data shows Rufinamide's higher retention in the GI tract due to liver metabolism, while brain uptake reflects BBB passage. The [^18^F]rufinamide shows uptake in the brain, but its concentration is likely low. The observed brain uptake indicates that [^18^F]rufinamide is able to cross the blood–brain barrier and is suitable for further evaluation as a PET imaging tracer. Overall, the data align with the pharmacokinetics of rufinamide, highlighting its absorption, metabolism, and selective distribution. A limitation of this study is the absence of *in vivo* metabolite identification, which will be addressed in future disease-model investigations.

## 4. Conclusion

In this study, the [^18^F]rufinamide was successfully synthesized as a radiotracer for epileptic brain imaging. The radiolabelling procedure was optimized under various conditions, including temperature, time interval and heating conditions. The labelling efficiency of the radiolabelled compound was found to be excellent in microwave reactor at 120 °C for 20 minutes in the second radiolabelling approach to give ∼20 ± 5% (decay corrected; 250–400 MBq). The labelled [^18^F]rufinamide screened physicochemical characterization, including partition coefficient (log *D*_7.4_) and percentage of plasma protein binding (%PPB). The log *D*_7.4_ shows 0.72 ± 0.03, which means the labelled radiotracer is lipophilic, and the %PPB in human serum shows 82.5 ± 2.1%. The SUV_max_ (lbm) graph illustrates the accumulation of activity in the brain of the normal rat, followed by rapid elimination over time. The clearance of the radiotracer through the GI and kidneys and its reach in the intestine and bladder, respectively, was observed within the 120 min scan. Overall, the data align with Rufinamide's pharmacokinetics, highlighting its absorption, metabolism, and selective distribution, as reported in the literature. These findings correlate with the known pharmacokinetic profile of rufinamide and provide foundational insights for its application in non-invasive imaging.

## Ethical approval

All animal procedures were performed in accordance with the Guidelines for the Care and Use of Laboratory Animals of Sanjay Gandhi Postgraduate Institute of Medical Sciences (SGPGIMS), Lucknow, India, and were approved by the Institutional Animal Ethics Committee (protocol no. 09-2018-2022).

## Author contributions

Vaibhav Pandey: methodology, investigation, formal analysis. Mohd. Faheem: validation, investigation. Sanjay Gambhir: validation, conceptualization, and Manish Dixit: supervision, funding acquisition, conceptualization.

## Conflicts of interest

The authors declare no competing interests.

## Supplementary Material

RA-016-D5RA09547F-s001

## Data Availability

The data supporting this article are included in the supplementary information (SI) file. Supplementary information: NMR spectra, LC-MS, radiochemistry, quality assurance, and PET scan data. See DOI: https://doi.org/10.1039/d5ra09547f.

## References

[cit1] Thijs R. D., Surges R., O'Brien T. J., Sander J. W. (2019). Epilepsy in adults. Lancet.

[cit2] Beghi E. (2020). The epidemiology of epilepsy. Neuroepidemiology.

[cit3] Duncan J. S., Sander J. W., Sisodiya S. M., Walker M. C. (2006). Adult epilepsy. Lancet.

[cit4] World Health Organization , Epilepsy, 2024, https://www.who.int/news-room/fact-sheets/detail/epilepsy

[cit5] Milligan T. A. (2021). Epilepsy: a clinical overview. Am. J. Med..

[cit6] Wier H. A., Cerna A., So T. Y. (2011). Rufinamide for pediatric patients with Lennox-Gastaut syndrome: a comprehensive overview. Pediatric Drug.

[cit7] Arroyo S. (2007). Rufinamide. Neurotherapeutics.

[cit8] Hakimian S., Cheng-Hakimian A., Anderson G. D., Miller J. W. (2007). Rufinamide: a new anti-epileptic medication. Expert Opin. Pharmacother..

[cit9] Sukprakun C., Tepmongkol S. (2022). Nuclear imaging for localization and surgical outcome prediction in epilepsy: A review of latest discoveries and future perspectives. Front. Neurol..

[cit10] Riondato M. and Eckelman W. C., Radiopharmaceuticals, in PET-CT and PET-MRI in Neurology: SWOT Analysis Applied to Hybrid Imaging, Cham: Springer International Publishing, 2016, pp. 31–57

[cit11] Hosford D. A., Crain B. J., Cao Z., Bonhaus D. W., Friedman A. H., Okazaki M. M., Nadler J. V., McNamara J. O. (1991). Increased AMPA-sensitive quisqualate receptor binding and reduced NMDA receptor binding in epileptic human hippocampus. J. Neurosci..

[cit12] Mukherjee J., Constantinescu C. C., Hoang A. T., Jerjian T., Majji D., Pan M. L. (2015). Dopamine D3 receptor binding of 18F-fallypride: Evaluation using in vitro and in vivo PET imaging studies. Synapse.

[cit13] Tipre D. N., Zoghbi S. S., Liow J. S., Green M. V., Seidel J., Ichise M., Innis R. B., Pike V. W. (2006). PET imaging of brain 5-HT1A receptors in rat in vivo with 18F-FCWAY and improvement by successful inhibition of radioligand defluorination with miconazole. J. Nucl. Med..

[cit14] Treyer V., Gietl A. F., Suliman H., Gruber E., Meyer R., Buchmann A., Johayem A., Unschuld P. G., Nitsch R. M., Buck A., Ametamey S. M. (2020). Reduced uptake of [11C]-ABP688, a PET tracer for metabolic glutamate receptor 5 in hippocampus and amygdala in Alzheimer's dementia. Brain Behav..

[cit15] Salmi E., Aalto S., Hirvonen J., Långsjö J. W., Maksimow A. T., Oikonen V., Metsähonkala L., Virkkala J., Någren K., Scheinin H. (2008). Measurement of GABAA receptor binding in vivo with [11C] flumazenil: a test–retest study in healthy subjects. Neuroimage.

[cit16] Barker J. S., Hines R. M. (2020). Regulation of GABAA receptor subunit expression in substance use disorders. Int. J. Mol. Sci..

[cit17] Tsartsalis S., Moulin-Sallanon M., Dumas N., Tournier B. B., Ghezzi C., Charnay Y., Ginovart N., Millet P. (2014). Quantification of GABAA receptors in the rat brain with [123I] iomazenil SPECT from factor analysis-denoised images. Nucl. Med. Biol..

[cit18] Salmi E., Aalto S., Hirvonen J., Långsjö J. W., Maksimow A. T., Oikonen V., Metsähonkala L., Virkkala J., Någren K., Scheinin H. (2008). Measurement of GABAA receptor binding in vivo with [11C] flumazenil: a test–retest study in healthy subjects. Neuroimage.

[cit19] Chugani D. C., Muzik O. (2000). α [C-11] Methyl-L-tryptophan PET maps brain serotonin synthesis and kynurenine pathway metabolism. J. Cereb. Blood Flow Metab..

[cit20] Arisawa T., Miyazaki T., Ota W., Sano A., Suyama K., Takada Y., Takahashi T. (2021). [11C] K-2 image with positron emission tomography represents cell surface AMPA receptors. Neurosci. Res..

[cit21] Everix L., Elvas F., Miranda Menchaca A., Khetarpal V., Liu L., Bard J., Staelens S., Bertoglio D. (2025). Preclinical validation and kinetic modelling of the SV2A PET ligand [18F] UCB-J in mice. J. Cereb. Blood Flow Metab..

[cit22] Verbeek J., Eriksson J., Syvänen S., Labots M., de Lange E. C., Voskuyl R. A., Mooijer M. P., Rongen M., Lammertsma A. A., Windhorst A. D. (2012). [^11^C] phenytoin revisited: synthesis by [11C]CO carbonylation and first evaluation as a P-gp tracer in rats. EJNMMI Res..

[cit23] García-Varela L., García D. V., Kakiuchi T., Ohba H., Nishiyama S., Tago T., Elsinga P. H., Tsukada H., Colabufo N. A., Dierckx R. A., van Waarde A. (2020). Pharmacokinetic Modeling of (R)-[11C] verapamil to Measure the P-Glycoprotein Function in Nonhuman Primates. Mol. Pharmaceutics.

[cit24] Jauhar S., Veronese M., Rogdaki M., Bloomfield M., Natesan S., Turkheimer F., Kapur S., Howes O. D. (2017). Regulation of dopaminergic function: an [18F]-DOPA PET apomorphine challenge study in humans. Transl. Psychiatry.

[cit25] Inoue Y., Abe O., Kawakami T., Ozaki T., Inoue M., Yokoyama I., Yoshikawa K., Ohtomo K. (2001). Metabolism of 99mTc-ethylcysteinate dimer in infarcted brain tissue of rats. J. Nucl. Med..

[cit26] Tiger M., Svensson J., Liberg B., Saijo T., Schain M., Halldin C., Farde L., Lundberg J. (2020). [11C] raclopride positron emission tomography study of dopamine-D2/3 receptor binding in patients with severe major depressive episodes before and after electroconvulsive therapy and compared to control subjects. Psychiatry Clin. Neurosci..

[cit27] McGinnity C. J., Hammers A., Barros D. A., Luthra S. K., Jones P. A., Trigg W., Micallef C., Symms M. R., Brooks D. J., Koepp M. J., Duncan J. S. (2014). Initial evaluation of 18F-GE-179, a putative PET Tracer for activated N-methyl D-aspartate receptors. J. Nucl. Med..

[cit28] Taylor N. J., Emer E., Preshlock S., Schedler M., Tredwell M., Verhoog S., Mercier J., Genicot C., Gouverneur V. (2017). Derisking the Cu-mediated 18F-fluorination of heterocyclic positron emission tomography radioligands. J. Am. Chem. Soc..

[cit29] Jacobson O., Kiesewetter D. O., Chen X. (2015). Fluorine-18 radiochemistry, labeling strategies and synthetic routes. Bioconjugate Chem..

[cit30] Attiná M., Cacace F., Wolf A. P. (1983). Displacement of a nitro-group by [18F] fluoride ion. A new route to aryl flurides of high specific activity. J. Chem. Soc. Chem. Commun..

[cit31] Haveman L. Y., Vugts D. J., Windhorst A. D. (2023). State of the art procedures towards reactive [18F] fluoride in PET tracer synthesis. EJNMMI Radiopharm. Chem..

[cit32] Padmaja R. D., Chanda K. (2018). A short review on synthetic advances toward the synthesis of rufinamide, an antiepileptic drug. Org. Process Res. Dev..

[cit33] Faheem M. , Pandey V. and Dixit M., [18F] Fluoro Analogue of D-Glucose: A Chemistry Perspective, In2-Deoxy-D-Glucose: Chemistry and Biology, Bentham Science Publishers, 2024, pp. 51–69

[cit34] Moore T. M., Akula M. R., Collier L., Kabalka G. W. (2013). A rapid microfluidic synthesis of [18F] fluoroarenes from nitroarenes. Appl. Radiat. Isot..

[cit35] Mandap K. S., Ido T., Kiyono Y., Kobayashi M., Lohith T. G., Mori T., Kasamatsu S., Kudo T., Okazawa H., Fujibayashi Y. (2009). Development of microwave-based automated nucleophilic [18F] fluorination system and its application to the production of [18F] flumazenil. Nucl. Med. Biol..

[cit36] Pandey S., Walia R., Kaur G., Pandav K., Rather I., Rana N., Sahoo S., Mittal B. R., Shukla J. (2025). Using the PET/CT radiotracer [68Ga] Ga-DOTA-mDesmo to target V1b receptors and localize corticotropinoma in Cushing's disease. Commun. Med..

[cit37] Taylor B. J., Orr S. A., Chapman J. L., Fisher D. E. (2009). Beyond-use dating of extemporaneously compounded ketamine, acepromazine, and xylazine: safety, stability, and efficacy over time. J. Am. Assoc. Lab. Anim. Sci..

[cit38] Sotoudeh N., Namavar M. R. (2022). Optimisation of ketamine-xylazine anaesthetic dose and its association with changes in the dendritic spine of CA1 hippocampus in the young and old male and female Wistar rats. Vet. Med. Sci..

