# Supplementary material for: Synthesis of [18F]rufinamide as a radiotracer for epileptic brain imaging
Source: RSC Adv. 2026 Mar 19;16(17):15350–60. doi: 10.1039/d5ra09547f (PMC13000467; doi:10.1039/d5ra09547f)
Supplement: RA-016-D5RA09547F-s001 [file RA-016-D5RA09547F-s001.pdf]

Supplementary Information (SI) for RSC Advances.
This journal is © The Royal Society of Chemistry 2026

Synthesis of [^{18}F]Rufinamide as a Radiotracer for Epileptic Brain Imaging

Vaibhav Pandey, Mohd. Faheem, Sanjay Gambhir, Manish Dixit*

Department of Nuclear Medicine, Sanjay Gandhi Postgraduate Institute of Medical Sciences, Lucknow, UP, India 226014.

Corresponding author: Manish Dixit; dixitm@sgpgi.ac.in

General Methods:

All solvents and reagents were purchased from Sigma-Aldrich and stored under dry conditions as mentioned on the product label. For the precursor synthesis, chemicals and solvents are stored in a desiccator under a nitrogen gas atmosphere. All reactions were carried out in oven-dried glassware. Fluorine-18 was obtained by irradiation of protons on [^{18}O]OH_2 (Huayi Isotopes) via the ^{18}O(p,n)^{18}F transformation using an 18 MeV Sumitomo Cyclotron (Sumitomo Heavy Industries Ltd, Japan). The irradiation was performed at a current of 30 µA for 10 minutes. At the end of bombardment, activity typically ranged from 5.55-9.25 GBq (150– 250 mCi). The radiolabeling was performed in DMSO as the solvent under various conditions, including different times, temperatures, and heating methods (conventional heating and microwave irradiation).

For the characterization of molecular structure, perform ^{1}H-NMR, ^{13}C-NMR and LC-MS spectra. For the NMR record, the compounds were dissolved in DMSO-d6 and scanned at 400MHz. The Mass data were recorded using an LC-MS (Agilent Technologies) system and analyzed on a sample without a column. All the compounds were dissolved in methanol and filtered through a 0.22 µm PVDF filter. The radiolabeling efficiency and radiochemical purity were calculated using radio thin-layer chromatography (rTLC; Miniscan Pro, Eckart & Ziegler Europe GmbH, Germany) and radio high-performance liquid chromatography (rHPLC, Agilent Technologies, Germany). All radiochemical yields were corrected to the [^{18}F]fluoride activity measured at the start of synthesis. The reaction conditions reported represent the highest yields obtained using manual radiosynthesis.

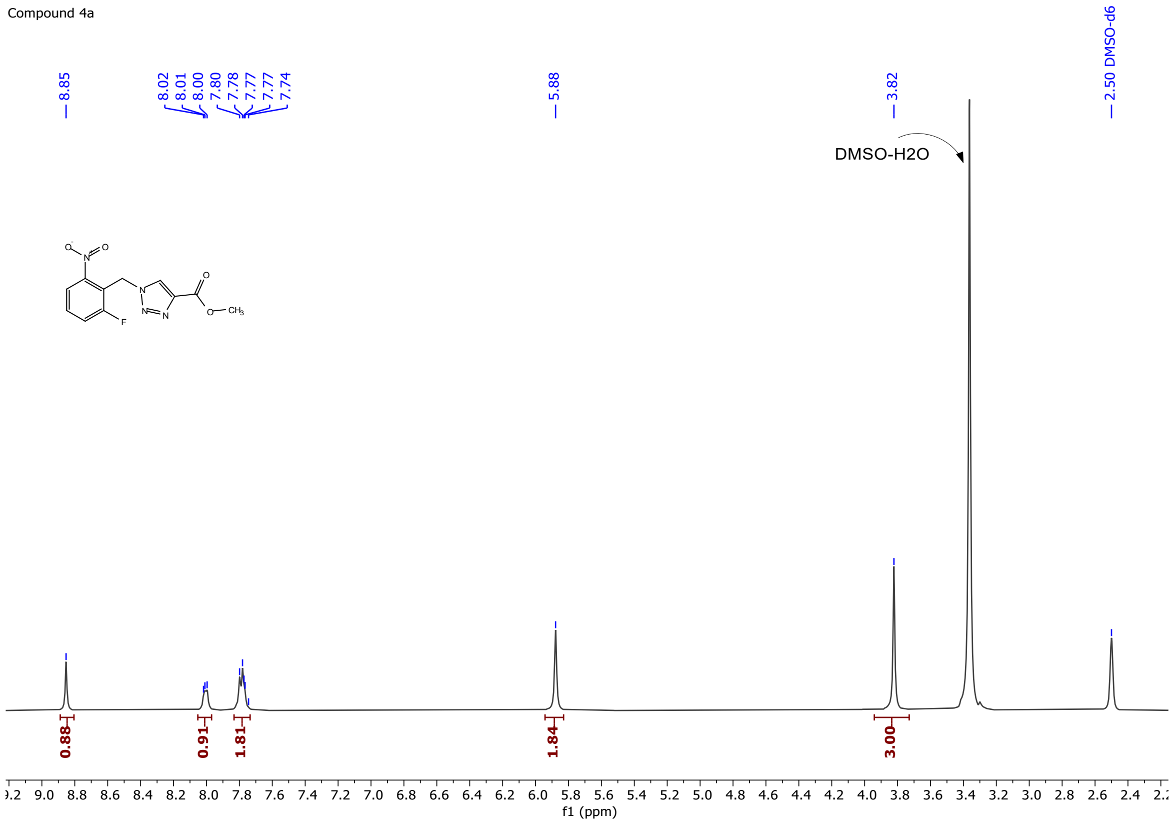

Figure 1: 1H-NMR of compound 4a *(methyl 1-(2-nitro-6-fluorobenzyl)-1H-1,2,3-triazole-4-carboxylate)*

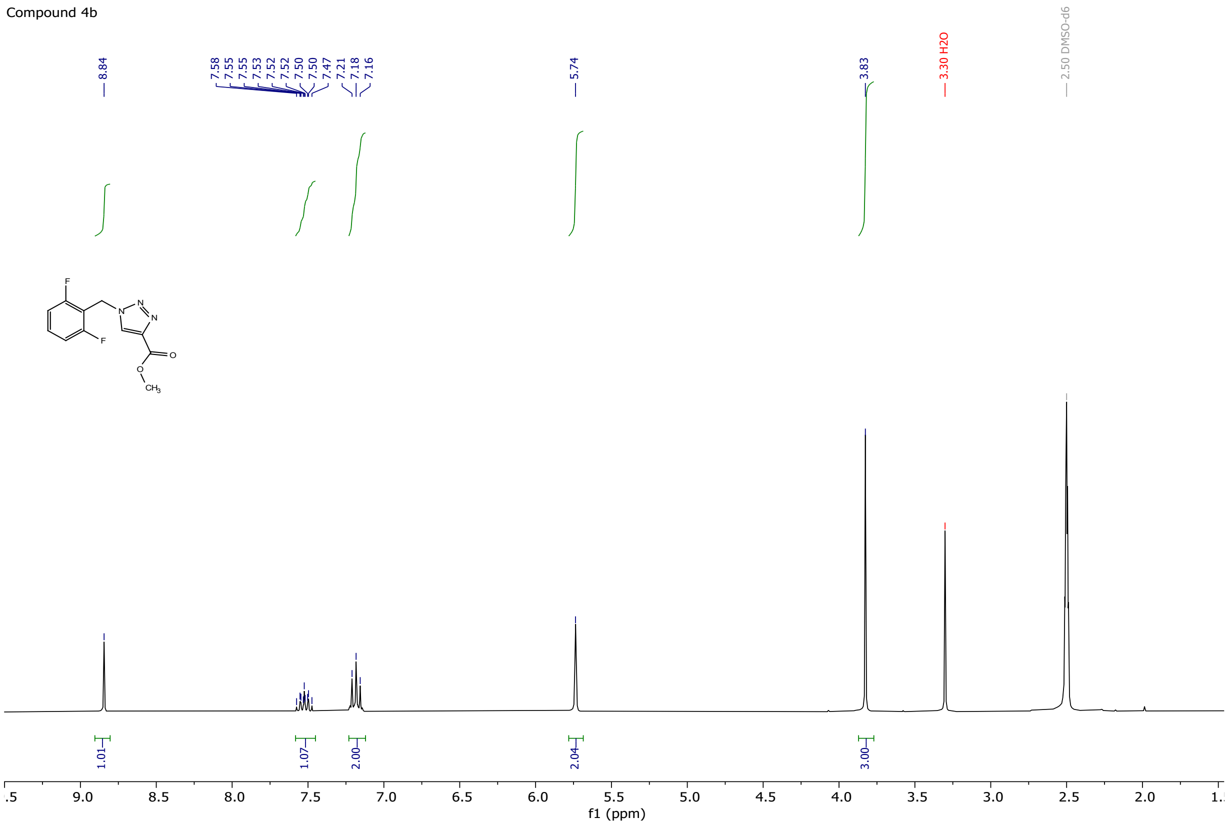

Figure 2: 1H-NMR of compound 4b *(methyl 1-(2-floro-6-fluorobenzyl)-1H-1,2,3-triazole-4-carboxylate)*

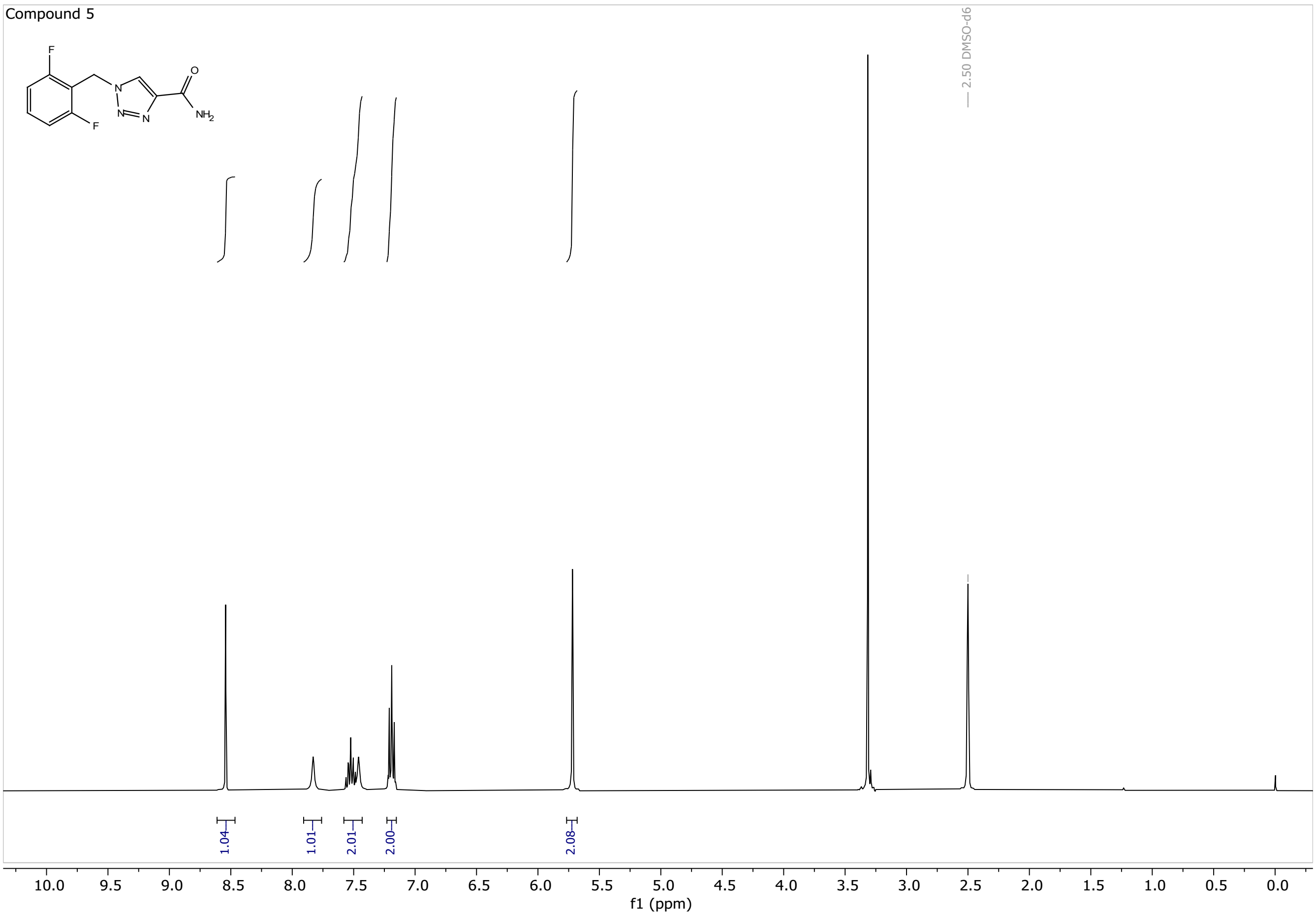

Figure 3: 1H-NMR of compound 5 *(1-(2,6-difluorobenzyl)-1H-1,2,3-triazole-4-carboxamide)*

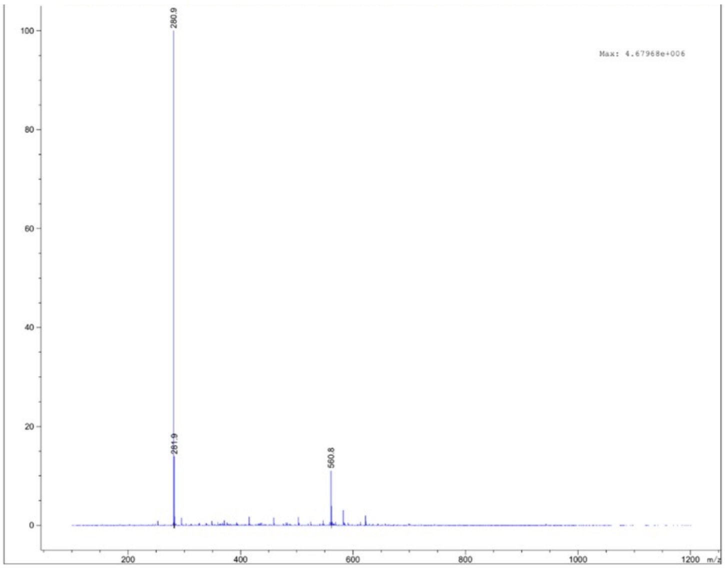

Compound 4a

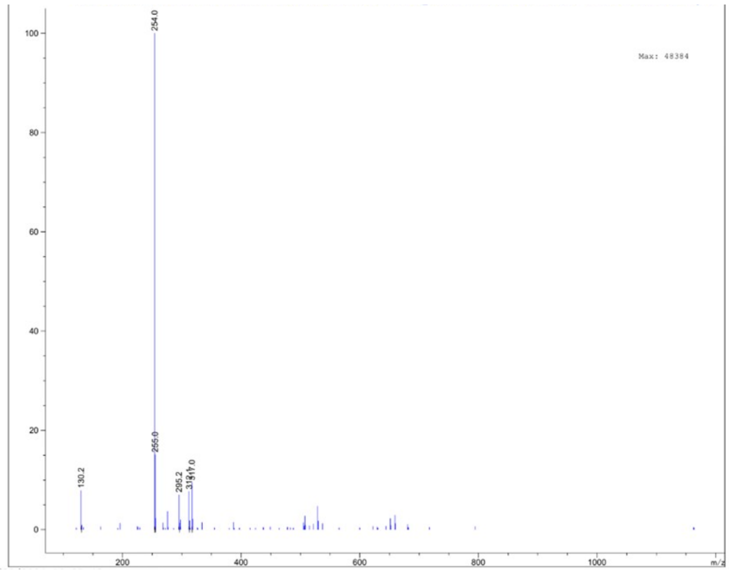

Compound 4b

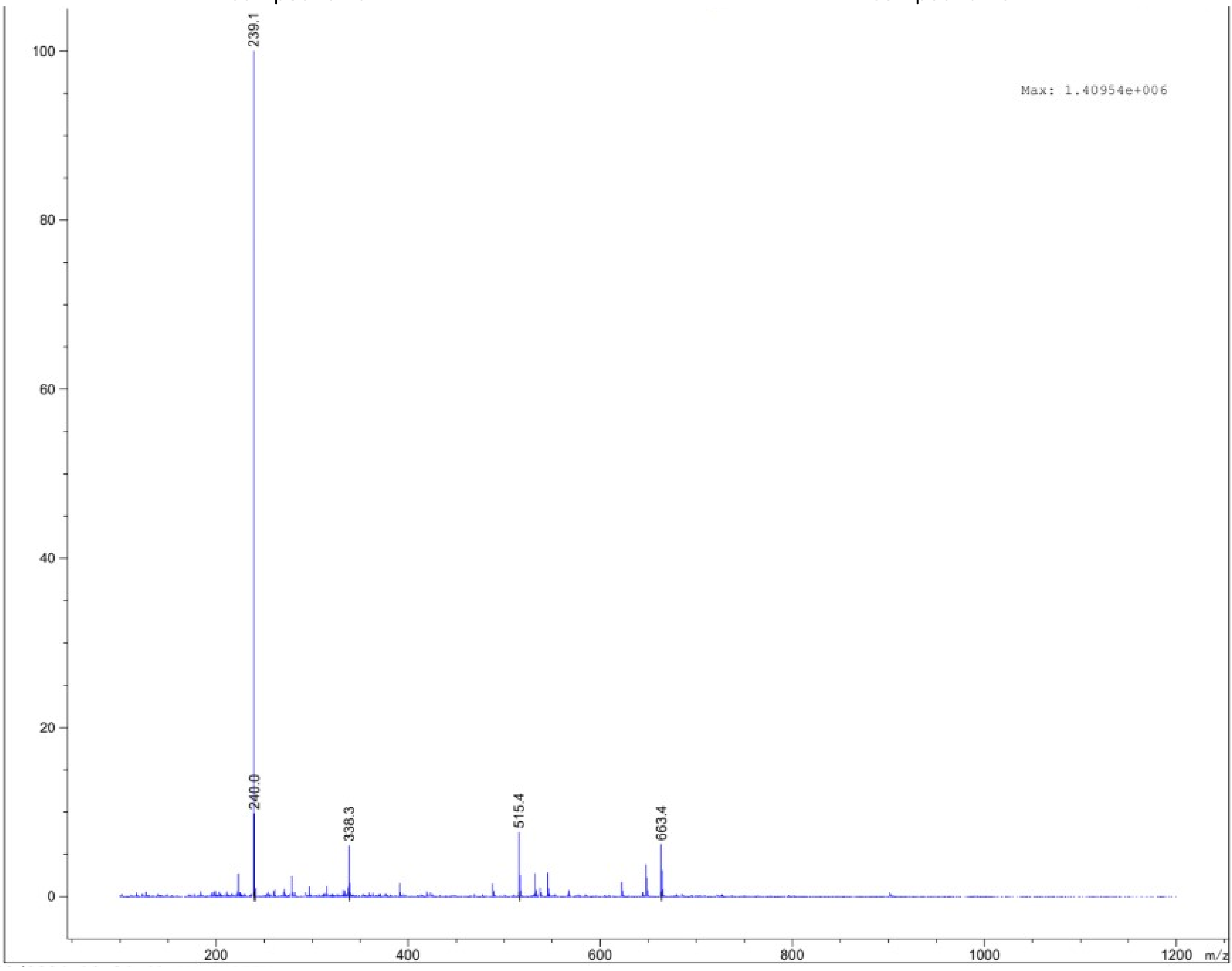

Compound 5

Figure 4: LC-MS (APCI+ESI) spectra of compound 4a, 4b and 5

Production of ^{18}F / Radiochemistry:

The production of 18F radioisotope via the Sumitomo 18MeV cyclotron in-house and collected directly on a pre-conditioned quaternary ammonium anion cartridge (QMA) via the CUPID system in CFN-MPS 100 (Sumitomo, Japan). The parameters of bombardment are current: 20mA, time: 10 min, and bombardment on 2.4mL [O-18}water (ABX Advance Med. Compound GmBH). The schematic workflow is as follows:

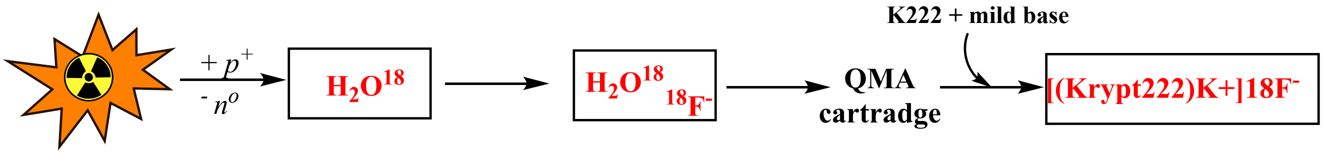

Figure 5: F-18 producton and purification

After collecting the ^{18}F-radioisotopes on a QMA cartridge, the ^{18}F was eluted by Kryptofix® (K222) with a mild base (K_2CO_3) in a V-shaped vial to form the ^{18}F-complex. The complex was dried by adding acetonitrile to form an azeotropic mixture at 85 ± 5°C under a nitrogen atmosphere. After drying, the ^{18}F complex was dissolved in an appropriate solvent for radiolabeling (dried DMSO) and radiolabeled with the precursor under different time intervals, temperatures, and thermal conditions. The labeling efficiency was determined by radio-thin layer chromatography (silica, 2 × 10 cm in size). The labelled and non-labelled counts were observed via miniscan rTLC. The labelling efficiency is as follows:

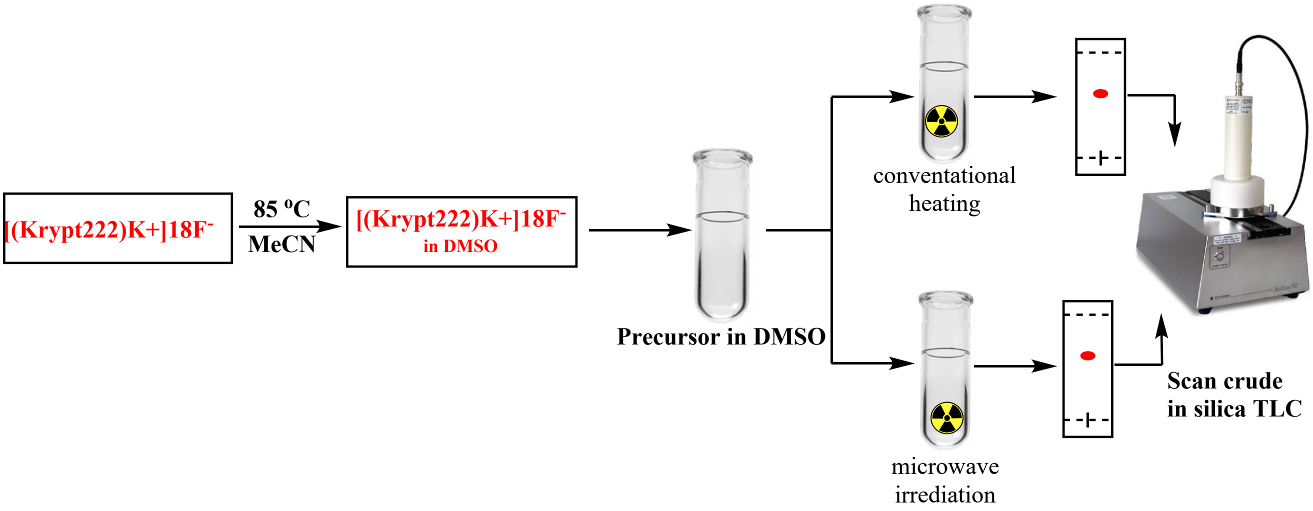

Figure 6: Radiolabeling and radiolabelling yield calcuation via rTLC.

The radiolabeled crude products are spotted on TLC and run in solvent (water: MeCN, 1:9). The counts were recorded and calculated labeling efficiency via labeled and non-labeled counts, which is listed below (in table 1, 2).

Table 1: In Microwave Irradiation condition:

Temp/time	3 min	6 min	10 min	15 min	20 min
80 ^oC	7.9±0.5	9.2±0.3	9.6±0.3	8.7±2.3	8.8±2.2
120 ^oC	15.3±0.6	19.9±1.6	25.6±1.0	28.9±1.3	51.8±8.5
150 ^oC	10.2±0.5	37.2±2.5	30.3±1.7	26.8±4.2	34.6±5.0

Table 2: In Conventional Heating condition:

Temp/time	3 min	6 min	10 min	15 min	20 min
80 ^oC	00	0	0	4.3±1.2	10.2±1.3
120 ^oC	8.6±0.6	13.2±1.7	11.6±2.4	16.8±3.1	18.4±1.7
150 ^oC	7.3±1.3	18.8±1.4	20.7±1.5	19.1±1.2	20.7±0.6

The radiolabeled crude product was purified by using a dried SePak C18 plus cartridge. The loading mixture should be a minimal quantity (200-500µL). After crude loading, the cartridge was washed with 20 mL of water to remove the non-labelled 18F fluoride. The purification was further confirmed by a radio-TLC scanner, which showed that after 20 mL of water washing, the ^{18}F fluoride counts peak was removed entirely as shown in Figure 7.

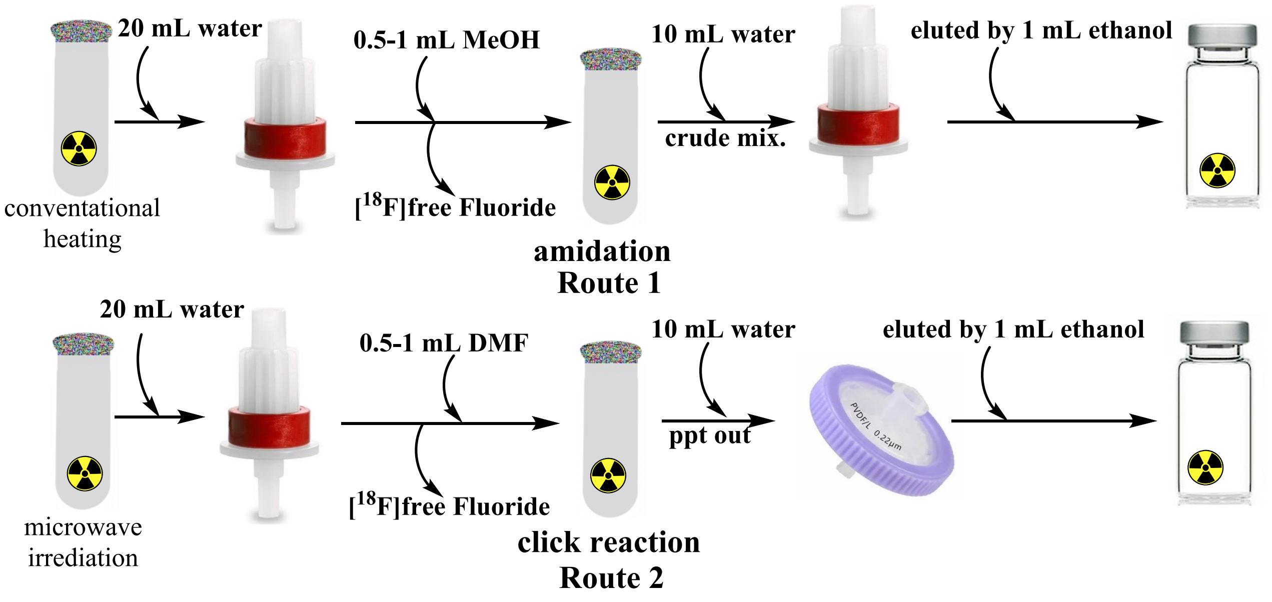

Figure 7: Representation of purification protocol of labellled compounds

The radioTLC graph for route one is attached below, where the red peak indicates the [^{18}F]fluoride ion peak and the yellow peak indicates the labelled peak (Figure 8). After purification, the compound was further processed for the following step reaction to achieve the targeted product.

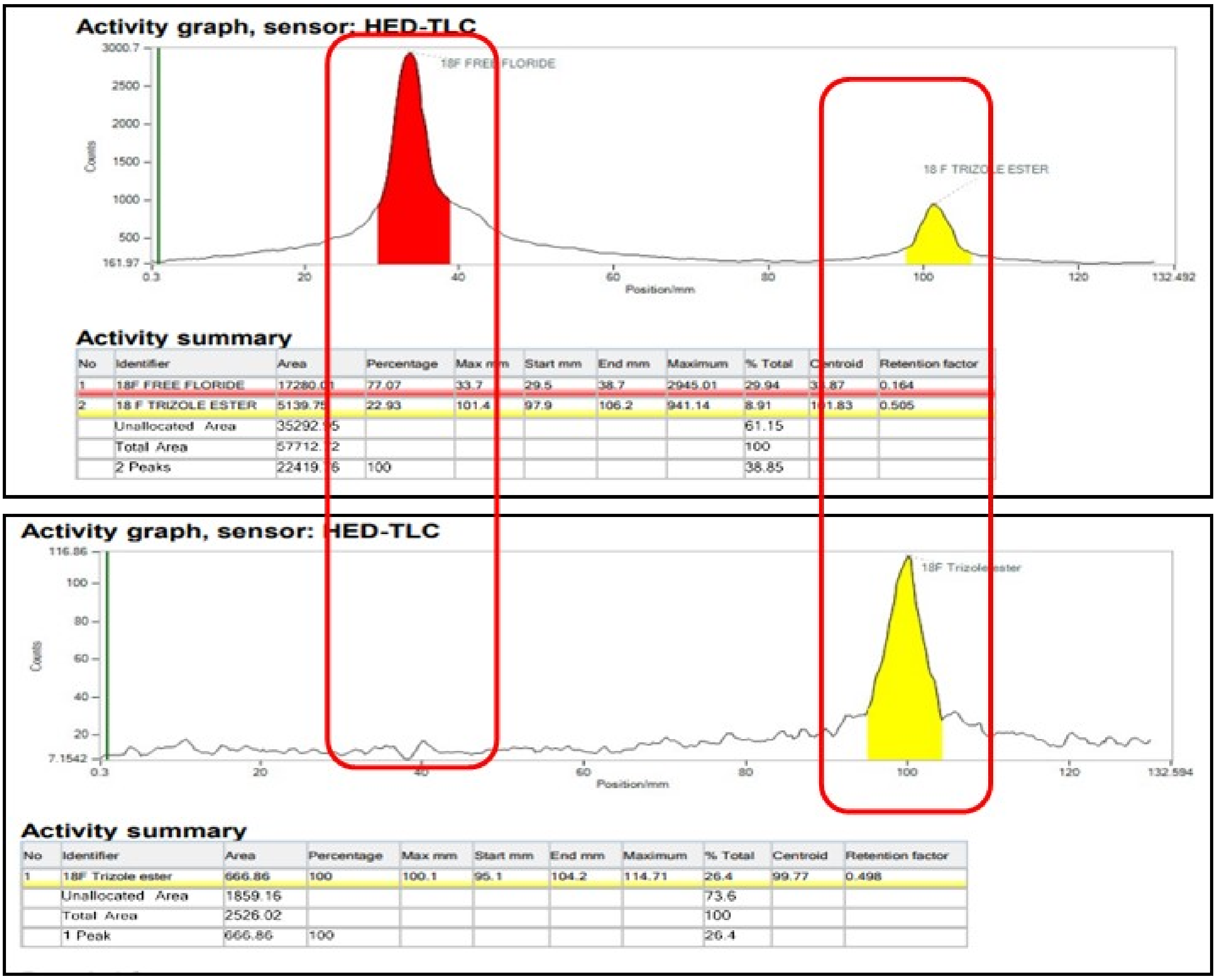

No	Identifier	Area	Percentage	Max mm	Start mm	End mm	Maximum	% Total	Centroid	Retention factor
1	18F FREE FLORIDE	17280.[illegible]1	77.07	33.7	29.5	38.7	2945.01	29.94	3[illegible].87	0.164
2	18 F TRIZOLE ESTER	5139.75	22.93	101.4	97.9	106.2	941.14	8.91	1[illegible]1.83	0.505
	Unallocated Area	35292.[illegible]5						61.15		
	Total Area	57712.[illegible]2						100		
	2 Peaks	22419.[illegible]6	100					38.85		

No	Identifier	Area	Percentage	Max mm	Start mm	End mm	Maximum	% Total	Centroid	Retention factor
1	18F Trizole ester	666.86	100	100.1	95.1	104.2	114.71	26.4	99.77	0.498
	Unallocated Area	1859.16						73.6		
	Total Area	2526.02						100		
	1 Peak	666.86	100					26.4		

Figure 8: rTLC radiogram of route 1

For route 2, the radio-TLC graphs are attached below. The colours peak indicates the same as explained above (Figure 9).

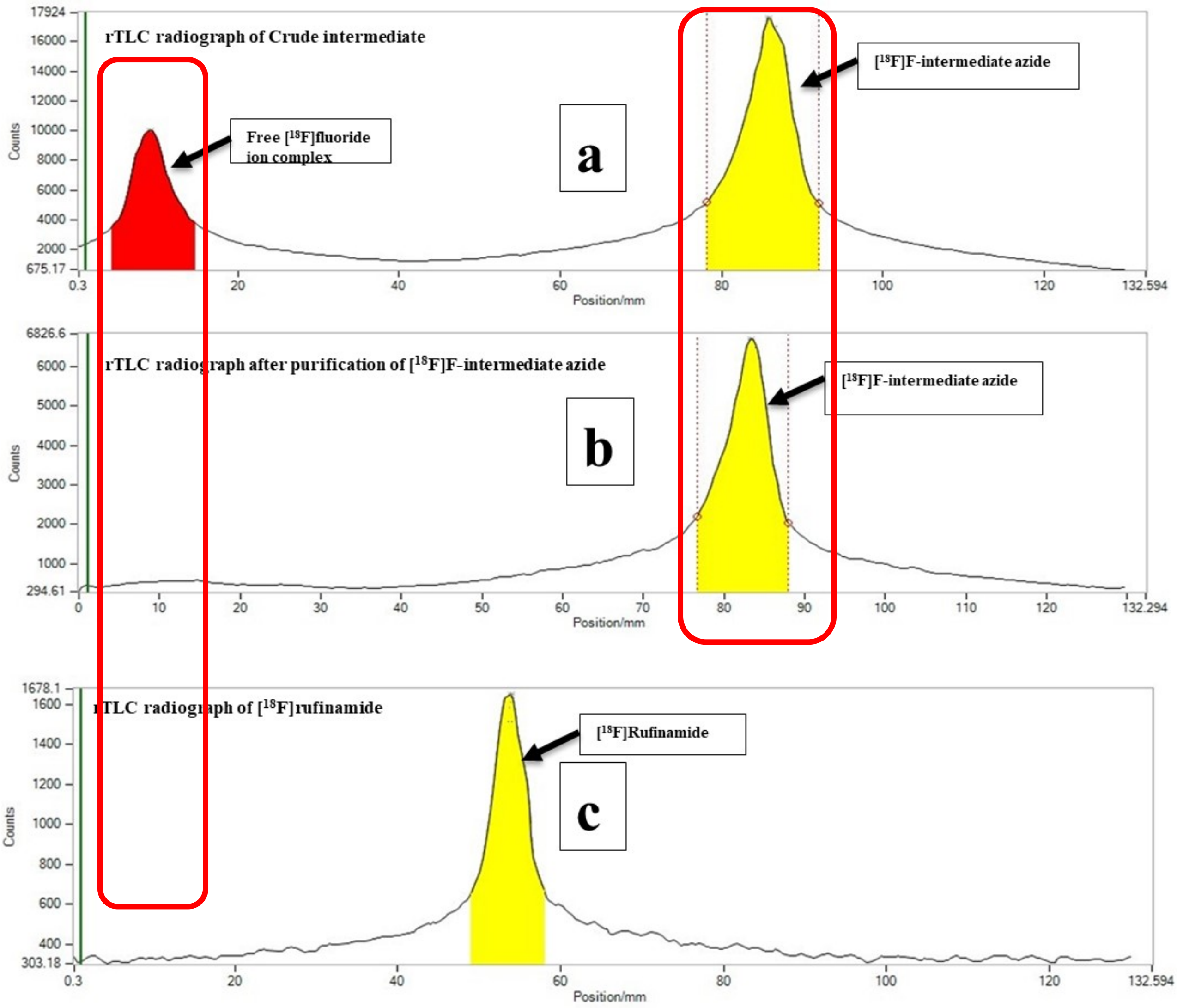

Figure 9: rTLC radiogram of route 2

After performing the second-step reaction, the radiolabelled compound was further verified for quality control via radio-high-performance liquid chromatography (rHPLC). The radio-HPLC spectra are attached to the manuscript, which show a purity of greater than 95%.

Calculation of $logD_{7.4}$:

The partition coefficient (logD7.4) of [^{18}F]Rufinamide was evaluated between n-octanol and phosphate-buffered saline (PBS, pH 7.4) and was determined. The 100µL of radiolabelled compound was added to a mixture of equal volumes of n-octanol and PBS (pH 7.4) in a centrifuge tube. The mixture was vortexed thoroughly for 15 minutes and centrifuged to separate the two phases. 100 µL of both the organic and aqueous layers were collected, and radioactivity counts were measured using a gamma counter (mentioned in table 3). The $logD_{7.4}$ value was calculated using the given formula.

$$logD = log_{10}(\frac{counts\ in\ octanol}{counts\ in\ PBS})$$

The counts of both media are as follows (Table 3):

Media	Gamma counts (Average)	Gamma counts (Average)	Gamma counts (Average)	
Octanol	5156981	4066797	4969859	
PBS, pH 7.4)	910256	803136	996585	
**$logD_{7.4}$**	0.753	0.704	0.697	0.72±0.03

% Plasma Protein Binding (PPB):

The plasma protein binding (%PPB) was determined by the precipitation method, as reported in the literature. The [^{18}F]Rufinamide compound (~100 µL; ~4MBq) was added and incubated with freshly collected human plasma (450 µL) and Albumin (450 µL) at 37°C for 60 minutes to allow protein binding equilibrium. After incubation, acetone was added to both mixtures to precipitate all the protein. The sample was subjected to vortexing and centrifuged for 6 minutes at 3,000g. The plasma protein is separated from the component. The radioactivity of both the filtrate (free fraction) and the precipitated sample was measured using a gamma counter (as shown in table 4). The %PPB was calculated using the formula:

$$\%PPB = (\frac{Pellet\ Count}{Supernatant\ Count + Pellet\ Count}) \times 100$$

Pellet count = bound fraction
supernatant count = free fraction

The count are as follows (Table 4):

Sample (%PPB)	*n=1*	*n=2*	*n=3*	Mean ± SD
**Albumin**	82.83	81.37	79.95	81.38 ± 1.17 %
**Human Serum**	84.81	80.99	81.57	82.46 ± 1.68 %

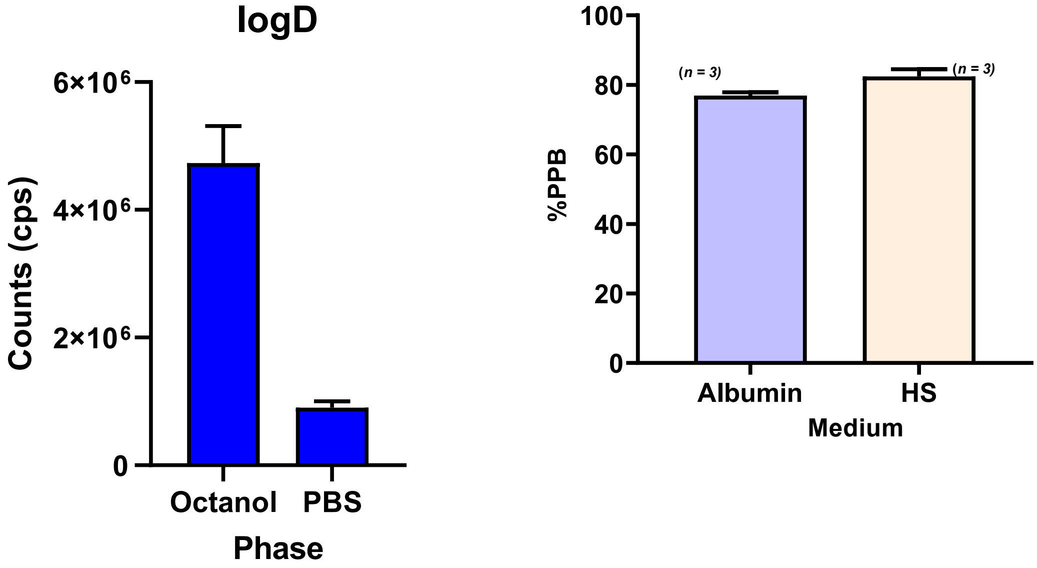

Figure 10. Physicochemical characterization of [18F]Rufinamide, including partition coefficient ($logD_{7.4}$) and plasma protein binding (%PPB) determined under physiological conditions. Data are presented as mean ± SD (*n = 3*).

PET/CT Scan of ^{18}F-Rufinamide: Biodistribution study

The PET/CT scan was performed using a Biograph mCT S(64) – 3R PET/CT scanner (Siemens Medical Solutions, USA, Inc.), and images were recorded at different time intervals. The PET/CT scan was set for 1.5 minutes per bed. A total of two BEDs were performed to complete the whole-body scan. The image files were processed using the Syngo.via software (Siemens Medical Solution, USA). In processing, the defined organ circle of 3D regions and Mean Standardized Uptake Values (SUV_{max} in activity/cm^3) were calculated. Excel generated the Organ SUV/Time-activity curves. The SUVmax at different time intervals (0–120 min) are as follows:

Table 5: Biodistribution data of [18F]Rufinamide

Organ	0 min	5 min	10 min	30 min	45 min	60 min	90 min	120 min
**Brain**	1.0	0.67	0.52	0.35	0.27	0.2	0.16	0.14
**Liver**	5.8	6.6	5.8	4.5	4.54	3.93	2.78	2.54
**Intestine**	1.4	1.25	1.3	1.2	23.2	33.1	16.1	10.1
**Kidney**	2.6	1.86	1.71	1.3	1.4	1.15	0.8	0.8
**Bladder**	2.04	7.8	9.54	18.46	22.7	24.5	24.4	27.1

Whole body scan with respective times are shown below:

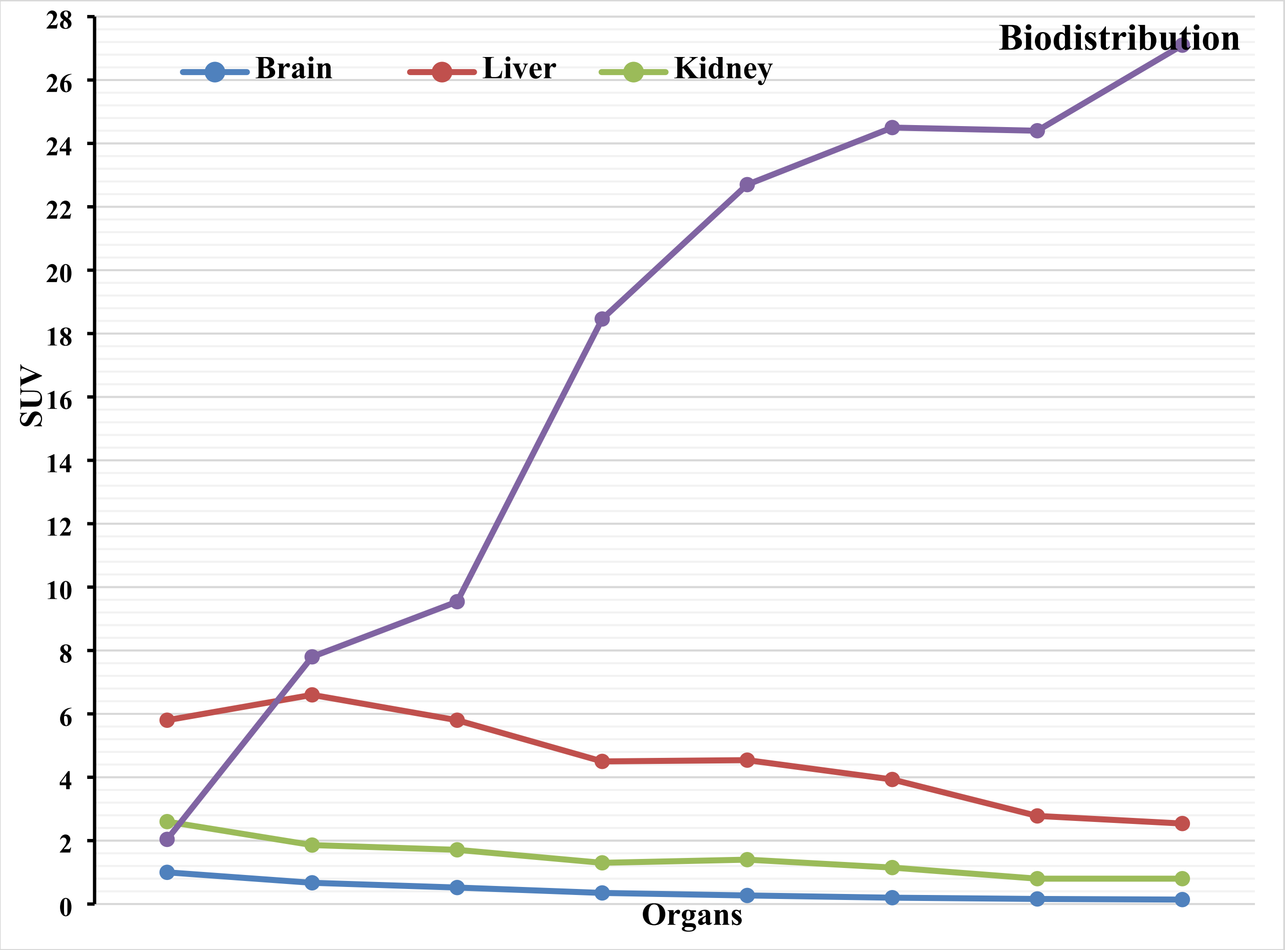

Figure 11: Target specific SUV graph

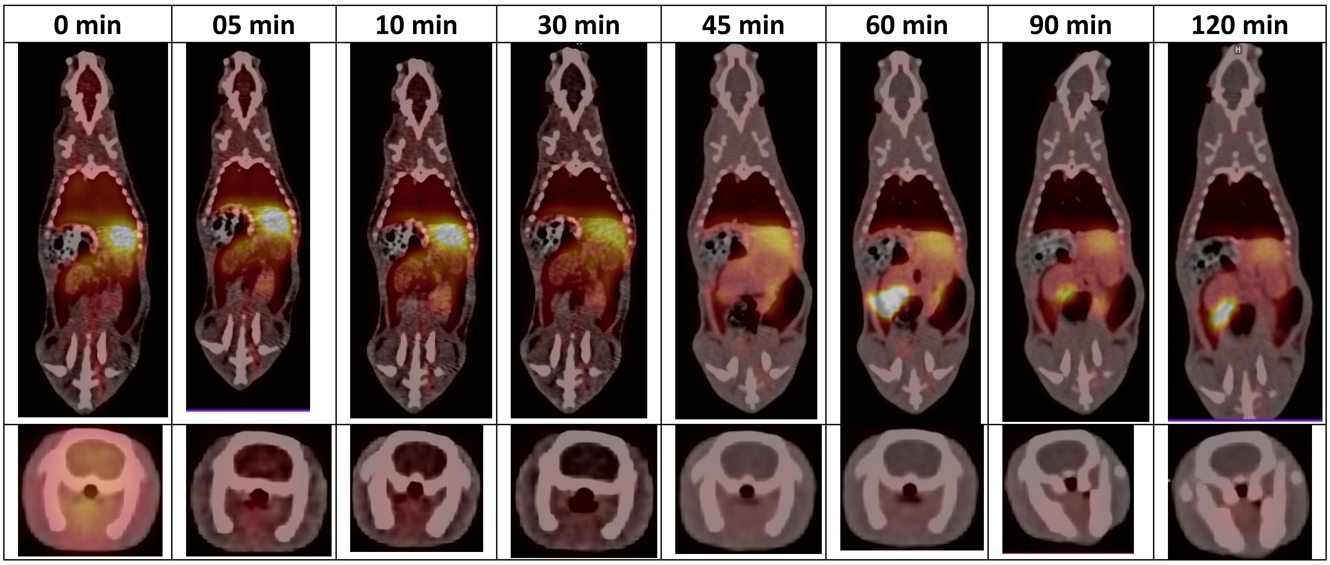

Figure 12: PET/CT data with different time interval

GI absorption

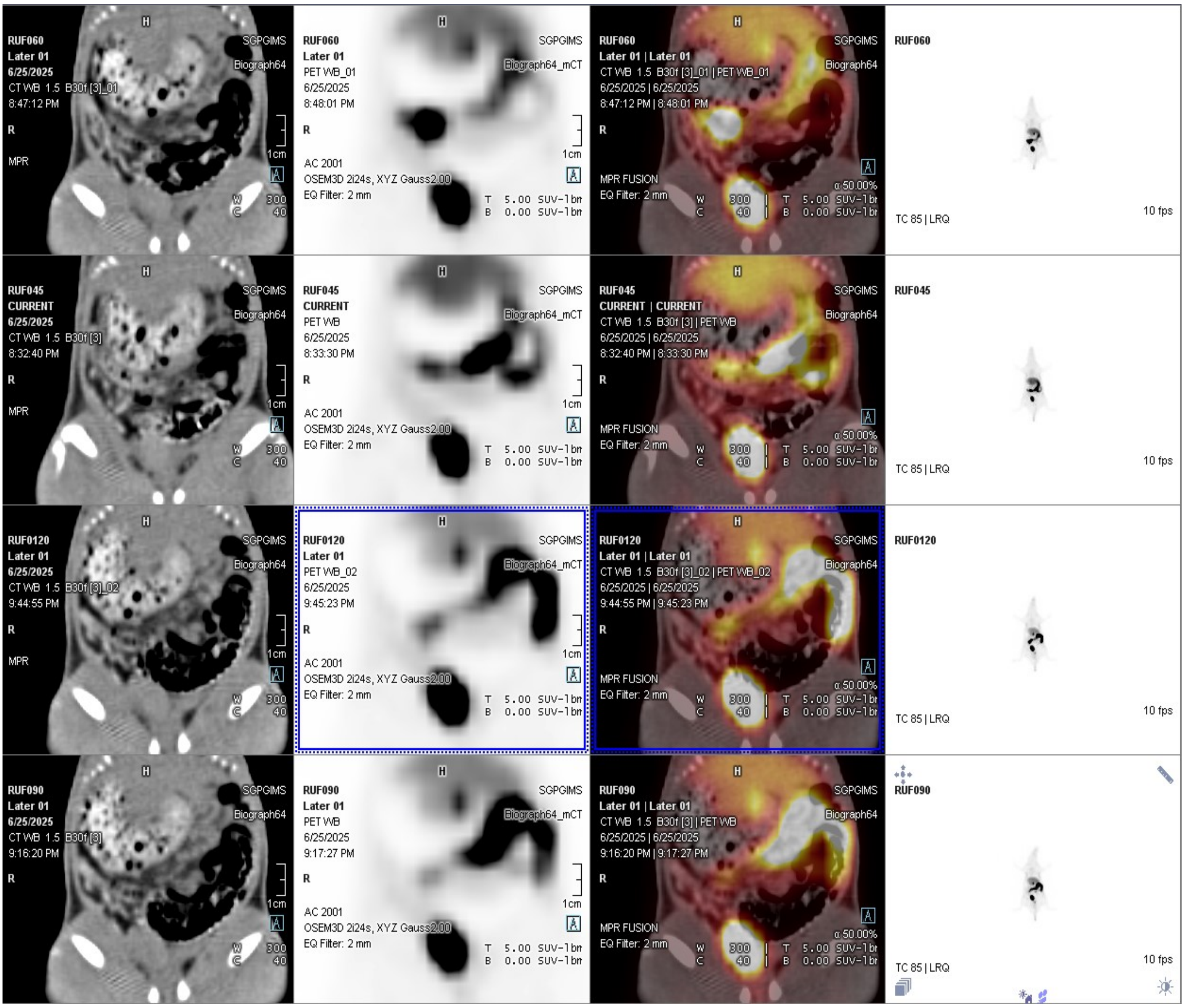

Figure 13: GI absorption with different time interval

Liver absorption

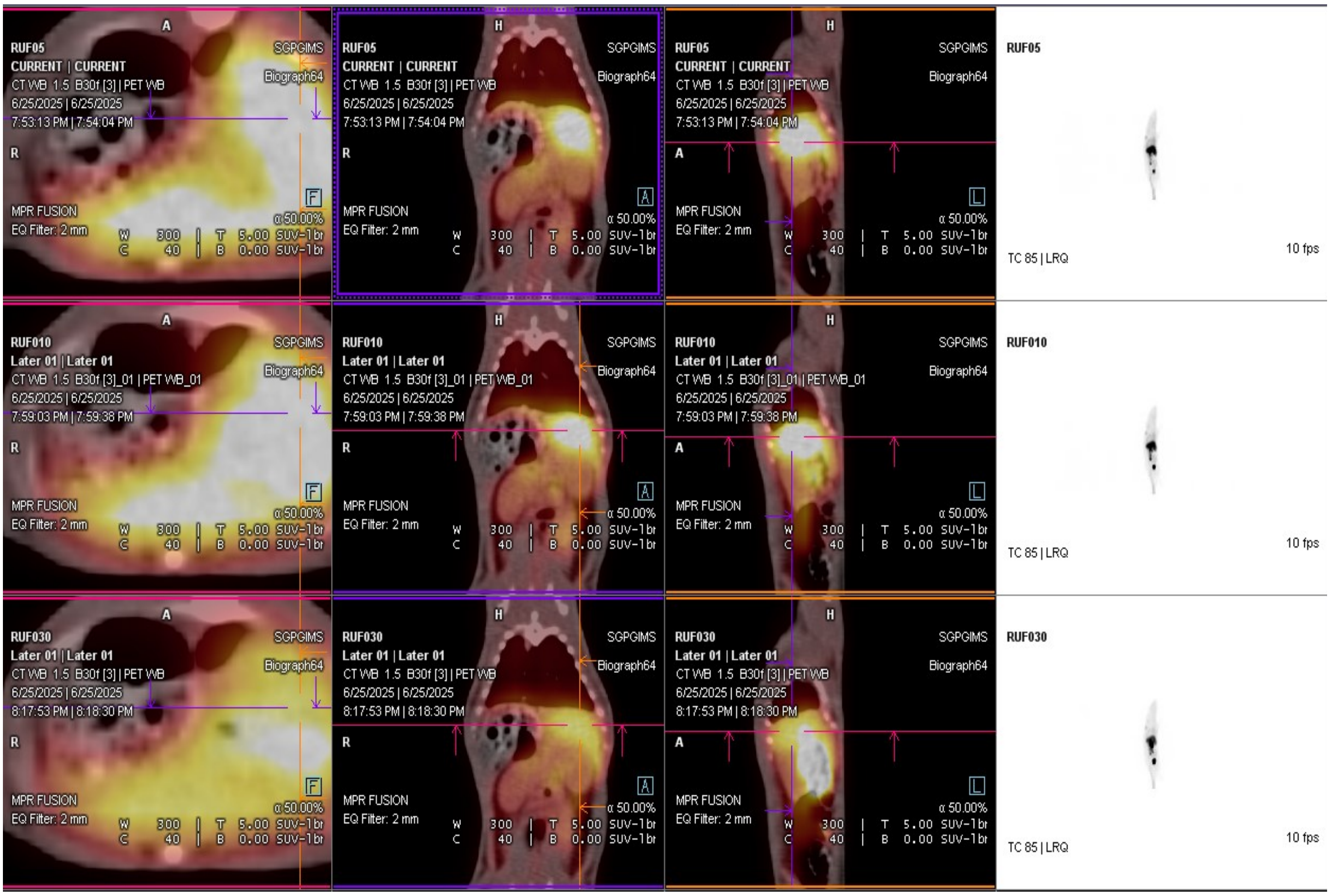

Figure 14: Liver absorption SUV with different time interval

Kidney absorption

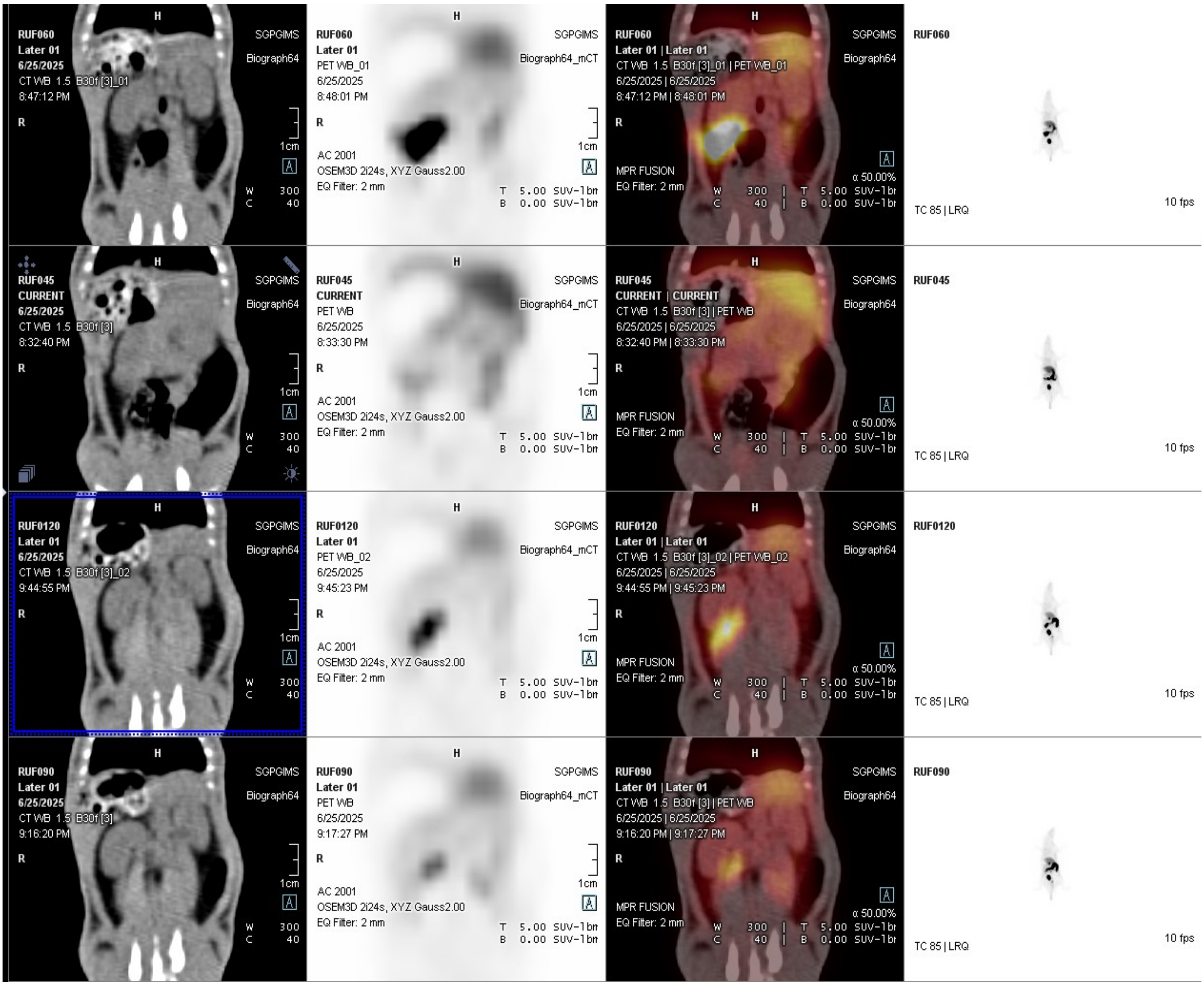

Figure 15: Kidney absorption SUV with different time interval

The following references support the pharmacokinetics of rufinamide.

1. Patsalos, P.N. (2016). Rufinamide. In: Antiepileptic Drug Interactions. Springer, Cham. https://doi.org/10.1007/978-3-319-32909-3_22
2. Perucca, Emilio, James Cloyd, David Critchley, and Eliane Fuseau. (2008). Rufinamide: clinical pharmacokinetics and concentration–response relationships in patients with epilepsy. *Epilepsia* 49, no. 7 : 1123-1141. https://doi.org/10.1111/j.1528-1167.2008.01665.x
3. Arzimanoglou, Alexis, Jose A. Ferreira, Andrew Satlin, Shannon Mendes, Betsy Williams, David Critchley, Edgar Schuck et al.(2016). Safety and pharmacokinetic profile of rufinamide in pediatric patients aged less than 4 years with Lennox-Gastaut syndrome: an interim analysis from a multicenter, randomized, active-controlled, open-label study." european *journal of paediatric neurology* 20, no. 3 393-402.https://doi.org/10.1016/j.ejpn.2015.12.015
